# Assessing dietary specialization to inform the conservation of the fairy pitta (*Pitta nympha*), an endangered vermivore

**DOI:** 10.7717/peerj.17189

**Published:** 2024-04-29

**Authors:** Jinseok Park, Jungmoon Ha, Woojoo Kim, Piotr G. Jablonski, Sang-im Lee

**Affiliations:** 1 Laboratory of Behavioral Ecology and Evolution, School of Biological Sciences, Seoul National University, Seoul, South Korea; 2 Museum and Institute of Zoology, Polish Academy of Sciences, Warsaw, Poland; 3 Laboratory of Integrative Animal Ecology, Department of New Biology, DGIST, Daegu, South Korea

**Keywords:** Conservation, Fairy pitta, Nestling diet, Vermivory, Earthworm, Home range, Diet specialist, Endangered species

## Abstract

Quantifying the diet of endangered species is crucial for conservation, especially for diet specialists, which can be more susceptible to environmental changes. The vulnerable fairy pitta (*Pitta nympha*) is considered a specialist that primarily feeds its nestlings with earthworms. However, there have been few studies of the nestling diet provisioned by parents, and no assessments of earthworm proportion in the diet of adults. Our study aimed to fill these gaps, shedding light on crucial factors for conservation. Combining new observations with existing literature, we confirmed a consistent dominance of earthworms in the nestling diet, regardless of rainfall, nestling age, and time of day. We extrapolated the total earthworm consumption during a breeding event, accounting for potential variation in the availability of earthworms and their prevalence in the adult diet. We used literature-based earthworm densities in pitta habitats and our estimates of family earthworm consumption to calculate the habitat area that could provide a pitta family with the number of earthworms consumed during a breeding event. The predictions matched observed pitta home range sizes when assumed that the adult diet is comprised of approximately 70% earthworms. The results highlight the importance of earthworm-rich habitats for conservation planning of the fairy pitta. To mitigate the effects of habitat destruction, we discuss conservation practices that may involve enhancing earthworm abundance in natural habitats and providing vegetation cover for foraging pittas in adjacent anthropogenic habitats rich in earthworms. To guide conservation efforts effectively, future studies should investigate whether previously reported breeding in developed plantation habitats is due to high earthworm abundance there. Future studies should also quantify correlations between local earthworm densities, home range size, and the breeding success of the fairy pitta.

## Introduction

Incorporating diet studies into conservation planning is crucial because the niche breadth of a species is associated with its capacity to adapt and adjust to environmental changes (Vázquez & Simberloff, 2002; Colles, Liow & Prinzing, 2009; Morelli et al., 2021). Diet specialists, defined as those with a narrow diet niche breadth (Irschick, Dyer & Sherry, 2005; Devictor et al., 2010; Morelli et al., 2021), are often found to be more vulnerable to change than dietary generalists. Consequently, specialists are more at risk of extinction than generalists (McKinney, 1997; Boyles & Storm, 2007; Clavel, Julliard & Devictor, 2011; Balisi, Casey & van Valkenburgh, 2018; Reed et al., 2020). Diet specialists are common worldwide, and for birds in particular, the degree of specialization strongly correlates with population decline (Morelli, Benedetti & Callaghan, 2020). Therefore, the concept of diet specialization should be considered when establishing priorities in conservation planning, such as determining which habitat to conserve or identifying the supplemental support a population may need.

**Figure 1:**
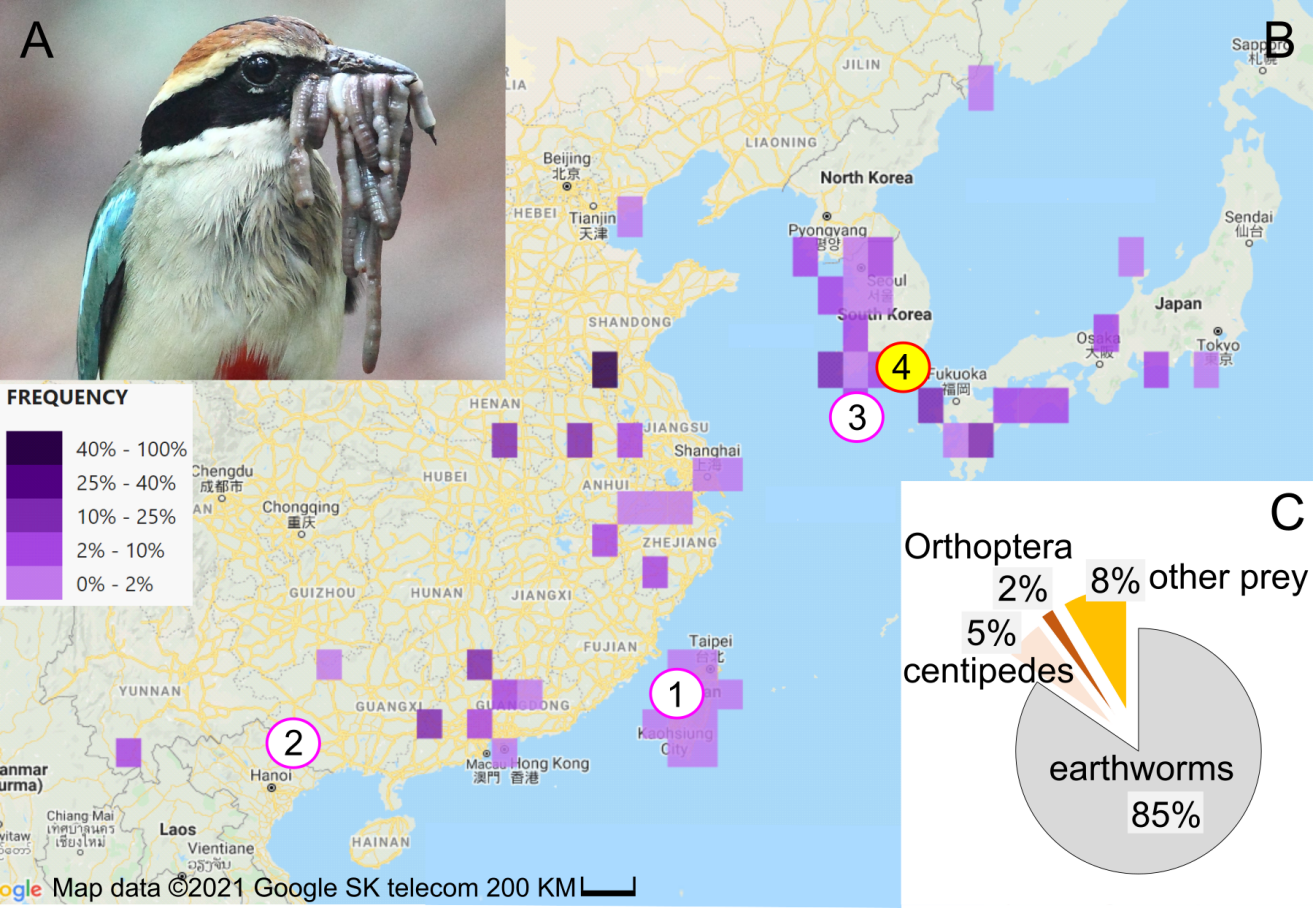
Study subject, study area, and diet composition. (A) Fairy pitta parent with a beak-load of earthworms, some of which are sundered (*i.e.,* cut into shorter pieces). Photo by JP. (B) Geographic distribution of fairy pitta observations during the breeding season (based on (The Cornell Lab of Ornithology, 2020) accessed on 1 June 2020), and the location of our study site (red circle no. 4 with yellow fill) relative to other sites (nos. 1, 2, 3) where data on nestling diet and parental care are available in the literature (pink circles, white fill): (1) Linnei, Taiwan (Lin, Yao & Lee, 2007), (2) Noongang, China (Jiang et al., 2017), (3) Jeju, Korea (Kim et al., 2012). (C) Diet composition at our study site based on a total of 647 prey items (details in Table S2). Map credit: 2021©Google SK telecom.

Among avian diet specialists, certain species, classified as vermivores, specialize in consuming earthworms during particular life stages. These species, belonging to families such as Pittidae, Grallaridae, Turdidae, and the genus *Scolopax*, primarily rely on worms during at least some stages, especially the breeding stage (Reynolds, Krohn & Jordan, 1977; Collar, 2020; Schulenberg & Kirwan, 2020; Winkler, Billerman & Lovette, 2020). The geographic distribution of Pittidae coincides with humid habitats, in which earthworms are relatively abundant (Lambert & Woodcock, 1996; Phillips et al., 2019). A total of 19 pitta species are near-threatened, vulnerable, endangered, or critically endangered (IUCN, 2022), including the vulnerable fairy pitta (*Pitta nympha*, Fig. 1A).

The fairy pitta breeds in Taiwan and southern China, Korea and Japan (Fig. 1B; Sullivan et al., 2009; The Cornell Lab of Ornithology, 2020; Erritzoe, 2020). Deforestation is the main threat to the species (BirdLife International, 2001; BirdLife International, 2017; BirdLife International, 2020), making habitat preservation a priority for conservation strategy. Fairy pittas prefer broadleaf forests (Lambert & Woodcock, 1996; BirdLife International, 2020; Erritzoe, 2020), but these habitats are being replaced by agricultural plantations. For example, bamboo plantations occupy over 50% of the area in Linnei, a breeding region in Taiwan (Lin, Yao & Lee, 2007). Habitat loss and fragmentation may reduce earthworm abundance, and thus lead to increased foraging distances (Séchaud et al., 2022) and home range size (Haskell, Ritchie & Olff, 2002) for vermivorous species. Preserving earthworm-rich habitats may therefore represent a conservation strategy that mitigates the fairy pitta’s population decline.

Previous quantitative studies on nestling diet (Lin, Yao & Lee, 2007; Kim et al., 2012; Jiang et al., 2017) consistently revealed earthworms as the primary dietary component, indicating that the species is a vermivore at least during the nestling stage. However, the proportion of earthworms in the adult diet is unknown. Furthermore, diet studies have historically been restricted to a small number of nests and small portion of the species’ geographic range (eight nests in Taiwan, one nest in SE China, and two nests in S Korea; Fig. 1B; Lin, Yao & Lee, 2007; Kim et al., 2012; Jiang et al., 2017). To establish an effective conservation plan for the species, more data are needed regarding the reliance of pittas on earthworms in different parts of their range and at different life stages. Particularly, quantifying earthworm consumption across a breeding event, by both the breeding pair and provisioned nestlings, could provide important insights into the role of earthworms in supporting pitta reproduction. Moreover, understanding how earthworm provisioning is influenced by time of day and nestling age could be crucial in mitigating the effects of human disturbance. This disturbance may cause longer incubation bouts, with the degree of this effect depending on the time of day (Steidl & Anthony, 2000). Disturbance can also cause a decrease of nestling growth, particularly during the later stages of development (Albores-Barajas, Soldatini & Furness, 2009). Finally, climate change is one of the primary factors relevant to the conservation of vulnerable species (Pearce-Higgins & Green, 2014). Understanding how diet composition varies with precipitation may provide insight into how changing weather patterns could affect the abundance, availability, and use of certain diet.

Here, we evaluate the diet composition of adult and nestling fairy pittas and assess the effects of nestling age, rainfall, and time of day on the proportion, length, and biomass of earthworms provided to nestlings (Pinkowski, 1978; Johnson & Best, 1982; Knapton, 1984). We then combine our results with data from past literature to assess the dependence of fairy pittas on earthworms across the species’ entire geographic range. We use the outcomes of these analyses to better understand the importance of earthworms in the diet of pittas and estimate the area of habitat that may be needed to support pittas through a breeding event. By comparing this estimate to the observed breeding home range sizes of fairy pittas, we extrapolate the importance of earthworms in the adult diet. Finally, we suggest conservation strategies depending on habitat fragmentation levels observed in satellite images of known geographical localities with breeding populations of the fairy pitta.

## Materials & Methods

### Ethics declarations

No animals were captured, harmed, or stressed in any way during this observational study. Observations were conducted from a distance without disturbance, in accordance with the laws of the Republic of Korea.

### Field work

Field work was previously described in Park et al. (2022). From May to July 2012, 2013, and 2017, we (JP) observed nestling provisioning at four fairy pitta nests in Namhae-gun in southern Republic of Korea (127°54′E, 34°50′N; no. 4 in Fig. 1B), which is the northern part of the pitta’s breeding range (Fig. 1B; The Cornell Lab of Ornithology, 2020; Erritzoe, 2020). We installed a dark-colored camouflaged tent 20 m from each nest and filmed and photographed visiting parents from the tent (Canon 1D Mark 4, Canon 7D, Canon 500D, lens Sigma 50–500 mm; Park, 2014). We removed the tent after the nestlings fledged. Observational details, such as the number of hours per nest or per day, are described in Table S1.

### Variables for statistical analyses

From videos and photos, we extracted the following variables for statistical analyses. Several variables listed below were also used in our earlier research (Park et al., 2022). Subsequent analyses using the extracted variables were performed on a subset of visits, excluding the first visit each day. This exclusion was necessary because the evaluation of the preceding inter-visit interval, an explanatory variable in multiple analyses, is not available for the first visits. Therefore, our sample sizes for analysis were as follows: 200 visits for all visits used in LMER/GLMER analyses, 192 visits containing earthworms, and 128 visits exclusively containing earthworms. The sample size for each analysis is also indicated in Table 1.

**Table 1:** Statistically evaluated models and ecological mechanisms. This table describes initial full models (left column) and the well-documented biological phenomena (right column) that may occur in the fairy pitta and may be responsible for the associations between the explanatory and response variables in the analyses. Due to our small sample size, we could not test all possible effects and interactions. In our observational data analysis, we use the information-theoretic approach, which helps determine mechanisms contributing more strongly to the observed pattern among mutually nonexclusive mechanisms. Please note that Analysis 1 and Analysis 5 are not independent of each other because Analysis 5 uses a subset of data used in Analysis 1, and the number of prey items (Analysis 1) is correlated with the number of earthworms (Analysis 5). The model specification uses variable names defined in the Methods section. The results are shown in Tables S4–S7 and S10–S11.

**Initial full statistical model: relationships evaluated in the model**	**Examples of multiple mutually non-exclusive mechanisms that may contribute to the relationships.**
Analysis 1 (Figs. 2B; S2; Table S4): Number of prey items ∼Earthworms present or only earthworms + inter-visit interval + nestling age class + time of day + (1—nest ID) Data set: All visits [n = 200 visits] or YesE visits [n = 192 visits]	1. The number of prey items will be larger in food loads containing earthworms (YesE, compared to NoE visits in the analysis using “earthworms present”, or OnlyE, compared to MIX in the analysis using “only earthworms”) because earthworms may be relatively easier to pack due to their softness compared to other prey with exoskeletons, wings, and legs. 2. The number of prey items may increase with longer inter-visit interval due to greater nestling hunger. Additionally, longer inter-visit intervals may indicate longer foraging trips, potentially leading to more prey collected. 3. Food loads for older nestlings may contain more prey because they require more food. 4. In the morning, food loads may contain more prey due to hungry nestlings, leading to increased foraging activity in adults and resulting in a higher number of prey items in a food load.
Analysis 2 (Fig. S3A; Table S5): Average earthworm length ∼ number of earthworms + nestling age class + time of day + inter-visit interval + (1—nest ID) Data set: OnlyE visits [n = 128 visits]	1. The average earthworm length in a food-load may decrease as the number of earthworms in the food-load increases due to beak size limits, assuming that birds tend to bring a full beak-load of food with each visit. 2-1. The average earthworm length delivered to older nestlings may be longer because they require more food and can swallow large prey. 2-2. The average earthworm length may not be associated with nestling age because parents might primarily follow optimal foraging rules rather than responding to changes in the size of their nestlings. 3. The average earthworm length in the morning may be longer than in the remaining times of the day because nestlings need more food, and birds can more easily find larger ones due to their putative higher availability on the soil surface in the morning compared to later in the day. 4-1. The average earthworm length may increase as the inter-visit interval increases because larger prey may require a longer time to catch and handle prey before bringing them to the nest. 4-2. The average earthworm length may not depend on the inter-visit interval, or it may decrease with an increase in the preceding inter-visit interval. This could happen if a long interval is an indicator of poor general earthworm availability, which in turn requires longer searching and causes parents to accept even smaller earthworms.
Analysis 3 (Figs. 2D, 2E; Table S6): Earthworms present or only earthworms ∼nestling age class + inter-visit interval + *time of day + (1—nest ID) * only used in models where “only earthworms” is the response variable. Data set: All visits [n = 200 visits] or YesE visits [n = 192 visits]	1. Visits with earthworms only (OnlyE type) may be more frequent for younger nestlings than for older ones because older nestlings require more food, and parents may include non-earthworm food items to deliver a greater amount of food to older nestlings. Additionally, very young nestlings may struggle to swallow harder prey in NoE visits, and parents may adjust the food type based on nestling age, delivering mostly earthworms (soft prey) to younger nestlings. 2-1. Longer inter-visit intervals may lead to less frequent OnlyE visits, as longer trips often indicate longer foraging trips, potentially resulting in encountering a more diverse range of prey by parents. 2-2. Feeding visit type may not necessarily be predicted based on the length of the inter-visit interval, especially if longer inter-visit intervals occur due to the extended handling time for longer prey (*e.g.*, sundering) or non-foraging activities. 3. OnlyE visits may be more frequent for nestlings in the morning than in the remaining times of the day because earthworm availability may be higher in the morning compared to later in the day (*e.g.*, due to hot and dry conditions later in the day).
Analysis 4 (Fig. S4; Table S10): Inter-visit interval ∼nestling age class + time of day + only earthworms + rainfall category + number of prey items + (1—nest ID). Data set: YesE visits [n = 192 visits]	1-1. The inter-visit interval may be shorter for older nestlings because they may require food more frequently to maintain their growth rate. 1-2. The inter-visit interval may be longer for older nestlings if parents aim at delivering larger food loads to larger nestlings (Analysis 7 addresses it) and if collecting a larger food load takes more time. 2. The inter-visit interval may be shorter in the morning than at other times of the day because nestlings are hungrier in the morning, necessitating more frequent food deliveries. This pattern is widespread among insectivorous birds. 3-1. The inter-visit interval for OnlyE visits may be shorter compared to MIX visits because the species specializes in hunting earthworms and may be less efficient at foraging for other prey. Other prey may be targeted only when earthworms are scarce, and it is time-consuming for birds to collect enough prey for the full beak-load of food. 3-2. The inter-visit interval for OnlyE visits may be longer because selectively seeking out earthworms could take more time, as they have to search specifically for this prey. 4-1. Heavy rain may increase the inter-visit interval because it may interfere with foraging and flight. 4-2. If heavy rain does not disrupt foraging activities, then rainy weather may shorten the inter-visit interval because earthworm availability is likely higher during rainy weather. 5. The inter-visit interval may be longer for visits involving a larger number of prey items because it takes more time to collect and handle a larger number of prey items.
Analysis 5 (Fig. 3A; Table S11): Number of earthworms ∼rainfall category + inter-visit interval + nestling age class + (1—nest ID). Data set: OnlyE visits [n = 128 visits]	1-1. Heavy rain may decrease the number of earthworms per visit because it may interfere with adult foraging behaviors. 1-2. Heavy rain may increase the number of earthworms per visit because it may increase earthworm activity on the surface, assuming that adult foraging behavior is not severely affected by heavy rain. 2. see no. 2 for Analysis 1 3. see no. 3 for Analysis 1
Analysis 6 (Fig. 3C; Table S11): Biomass of a single earthworm ∼rainfall category + inter-visit interval + nestling age class + (1—nest ID/visit ID). Data set: OnlyE visits [n = 328 earthworms within 128 visits]	1-1. Heavy rain causes more earthworms to emerge, increasing their availability, including larger worms, which are likely preferred by birds. Additionally, birds may rely on sound cues to detect worms, but wet litter reduces the sound created by movements, making larger worms the only ones detectable. 1-2. Heavy rain may hinder foraging and prey handling, especially for larger worms, potentially reducing the size of an average single earthworm in a food-load. 2. Finding a large earthworm may require longer foraging time compared to finding worms of any size, which could result in a longer inter-visit interval and be associated with heavier earthworms brought to the nest. 3. Food-loads brought to older nestlings may contain heavier earthworms than those brought to smaller nestlings because older nestlings require more food and can swallow larger items more easily.
Analysis 7 (Fig. 3E; Table S11): Biomass of all earthworms per visit ∼rainfall category + inter-visit interval + nestling age class + (1—nest ID). Data set: OnlyE visits [n = 128 visits]	If birds maximize the food-load per visit, the biomass of earthworms per OnlyE visit may be not affected by the factors in the model. However, there is also a possibility that: 1. Heavy rain may increase the total biomass per visit because earthworm availability is likely higher during/after rain. 2. With longer inter-visit intervals, parents likely collect more food. Additionally, the longer the nestlings wait for food, the more food they require. 3. The total biomass per visit is expected to be larger for older nestlings because they require more food.

**– Nest ID**: Each nest was given a unique ID. Since we observed four nests over three years, the variation among nests also represents variation among years.

**– Visit ID**: Across all four nests, each visit of a pitta parent to the nest was assigned a unique ID.

**– Time of day**: The time of each observed visit was categorized as morning (8:00 to 10:00 h), noon (10:00 to 14:00 h), or afternoon (14:00 to 18:00 h).

**– Inter-visit interval:** This refers to the duration in minutes between a parent leaving the nest and returning with food. When used as an explanatory variable in models analyzing visit-related characteristics, we considered the duration of the interval preceding the visit. We did not calculate the frequency of visits because the start and end times for each observation session were determined by the first and last observed visit rather than independently determined start/end times.

**– Prey type**: Whenever possible, a prey item was classified into one of 12 taxonomic categories (from genus to class level; Table S2) and two additional categories: unidentifiable arthropod and unidentifiable non-earthworm prey. In certain cases (*n* = 30), we were unable to determine the prey type due to poor lighting or its position in the beak. These data were excluded from statistical analyses regarding diet composition.

**– Earthworms present** (binary variable): Each feeding visit was categorized as either a visit with a food-load containing earthworms (YesE) or without earthworms (NoE).

**– Only earthworms** (binary variable): We subdivided the YesE category of the “Earthworms present” variable into two additional categories: only earthworms (OnlyE) or mixed food-load (MIX).

**– Number of prey items**: This represents the number of prey items per visit (per food-load brought at a visit).

**– Number of earthworms**: This indicates the number of earthworms per visit (per food-load).

**– Prey length:** The long axis length of each individual prey item was estimated from photographs and videos in units of beak length (to the nearest 0.25 beak length) and then converted it to centimeters, assuming a beak length of 2 cm (range: 1.90 to 2.06 cm, based on measurements from 46 specimens conducted by Lin, Yao & Lee, 2007). In total, 13 earthworms (distributed among nests as 7, 1, 2, and 3 earthworms) could not be measured due to poor lighting conditions or their position within the beak. These data were excluded from statistical analyses regarding prey length. Parents often sundered prey, primarily earthworms, into pieces (Park et al., 2022). To determine the length of the prey before sundering, we identified its color, thickness (diameter), and segment patterns (size and location) at the sundered point for each piece. Using these characteristics, we subsequently matched pieces to each other to determine those originating from the same earthworm.

**– Average earthworm length**: This represents the average length (cm) of earthworms in a prey load, calculated only for feeding visits containing only earthworms (OnlyE).

**– Biomass of a single earthworm** (in grams of ash-free dry mass, AFDM): We calculated AFDM of each earthworm from its estimated length using allometric equations (AFDM (g) = exp {3.19 × ln [length (mm)] − 15.85}) proposed by Greiner, Costello & Tiegs (2010) for Megascolecidae, the dominant earthworm fauna in South Korea (Blakemore, 2014; Hong & Csuzdi, 2016). Subsequently, we estimated the fresh mass of the earthworms by multiplying AFDM by 5.7904 (Onrust, 2017), which is similar to the ∼5.6 suggested by Reynolds (1972).

**– Biomass of earthworms per visit** (in g): We used the equation provided above to calculate the total earthworm biomass for each feeding visit containing only earthworms (OnlyE).

**– Nestling age class**: We classified nestling age as either “young” (1–7 day old brood) or “old” (8–13 day old brood). We used this categorical variable due to uneven representation of ages across the four nests (*i.e.,* the age of 13 days is not represented in any nest, age 1 day is represented in only one nest, ages 2, 6, and 12 days are presented in two nests, ages 3, 5, 8, and 11 days are represented in three nests, and only ages 4 and 9 days are presented in all four nests). Moreover, the effect of age on certain dependent variables, such as prey quantity or size, may be non-linear. This non-linearity could complicate the analysis, further compounded by the incomplete sampling of each of the 13 age levels based on one-day resolution. Considering that each nest contributes 2–7 days of sampling or 3–5 days of sampling to the respective two age categories (Table S1), using the categorical variable offers a more reliable statistical estimation. We also conducted an additional analysis (Fig. S1), which explores the effect of nestling age at the one-day resolution scale (with hatching day coded as day 1) on the probability of a specific visit type, offering hints into the shape of this relationship (Fig. S1). This detailed evaluation is impossible when age is coded as a two-level variable.

**– Rainfall category**: The daily cumulative precipitation (mm) on the day of observation was categorized as “light rain” for precipitation below 4 mm/day, and as “heavy rain” for precipitation exceeding 4 mm/day.

### Statistical analyses

#### General

We primarily conducted generalized (GLMER) or linear mixed-effects models (LMER) to assess the prevalence of earthworms in the provisioned nestling diet and evaluate the effects of rain, time of day, and nestling age on parental provisioning. As our data are purely observational and the multiple effects considered are not mutually exclusive, the classical null hypothesis-based approach is not advisable (Stephens et al., 2005; Richards, Whittingham & Stephens, 2011; Symonds & Moussalli, 2011; Aho, Derryberry & Peterson, 2014). Therefore, we adopted the information-theoretic framework. As is common in studies of threatened species, our small sample size (*n* = 4 nests) limited the analyses we could conduct for each dependent variable. This also meant we could not explore interactions between fixed effects. While our observational study may not offer sufficient data to distinguish between non-exclusive mechanisms, the information-theoretic approach allowed us to assess the degree to which our data support each of several mechanisms that may shape the role of earthworms in parental provisioning. A brief overview of all potential mechanisms and the corresponding statistical models used to evaluate these mechanisms is presented in Table 1.

#### Nestling age and time of day

Since young nestlings may struggle to swallow harder non-earthworm prey and older nestlings require more food, we predicted that the frequency of earthworms (*vs.* other prey types), visits with earthworms (*vs.* visits without earthworms), and visits containing only earthworms (*vs.* visits with a mixed food load) in the diet would be higher for younger nestlings. Considering that nestlings require more food in the morning and during the late stage of development, we further predicted higher frequencies of earthworm-containing feeding visits, longer average earthworm lengths, higher prey numbers, and shorter inter-visit intervals during these specific periods. Detailed predictions and alternative scenarios are presented in Table 1.

To determine how parental provisioning behavior and the contribution of earthworms to the provisioned nestling diet change with nestling age class and time of day, we included these two explanatory variables, along with other potentially associated explanatory variables, as fixed effects in GLMER or LMER models. Separate analyses were performed for the following response variables: earthworms present (binomial, logit link), only earthworms (binomial, logit link), number of prey items (Conway–Maxwell–Poisson, log link), average earthworm length (square-root transformed), and the inter-visit interval (square-root transformed).

In addition to the main analyses, we used a simple classical contingency table approach to examine the effect of nestling age class on both the frequency of earthworms and visits exclusively involving earthworms in the nestling diet. We carried out Fisher exact tests for each nest, followed by a Fisher’s combined probability test using the “survcomp” package (Whitlock, 2005). Here, other factors were not taken into consideration.

#### Rainy weather

To investigate how rainfall affects earthworm provisioning, we included rainfall as a fixed effect in GLMER or LMER models, along with other potentially important explanatory variables. For the analyses, we used the OnlyE (visits containing only earthworms) dataset, and the response variables were number of earthworms (Conway–Maxwell–Poisson, log link), biomass of a single earthworm (box-cox transformed; exponent value of 0.1), biomass of earthworms per visit (square-root transformed), and inter-visit interval (square-root transformed). Earthworms emerge from the soil during rainy weather due to the reduced risk of desiccation and decreased oxygen availability caused by heavy rain (Chuang & Chen, 2008). As a result, earthworm availability increases (Martay & Pearce-Higgins, 2018). We therefore expected that rainfall leads to increased provisioning with earthworms for nestlings, in terms of both quantity and quality.

#### Details of the LMER/GLMER analyses

In the examination of normality for residuals in our linear mixed-effects models (LMER), the Shapiro–Wilk test was employed. If the results did not meet the normality assumption, we applied transformations to the response variable to improve residual normality. Our initial approach involved a log transformation, and if this did not yield the desired improvement, we explored a square-root transformation. If neither transformation improved normality, a box-cox transformation was employed. LMER was conducted using the lmer function from the “lmerTest” package (Kuznetsova, Brockhoff & Christensen, 2017).

For generalized linear mixed-effects models (GLMER), particularly in relation to binary response variables such as earthworms present and only earthworms, we adopted the Binomial distribution with the logit link function. Regarding count-based response variables, such as the number of prey/earthworms, we opted for the Conway–Maxwell–Poisson distribution with the log link function. GLMER with binomial distribution was generated using the glmer function from the “lme4” package (Bates et al., 2015), while GLMER with Conway–Maxwell–Poisson distribution was performed using the glmmTMB function from the “glmmTMB” package (Brooks et al., 2017).

We used nest ID as a random effect to attempt generalizations to a population of nests. We also used visit ID (nested within nest ID) as a random effect in mixed models when appropriate. According to some opinions (Harrison, 2015), using nest ID as a random effect may pose problems since it only contains four levels. In response to this concern, we conducted a parallel analysis treating nest ID as a fixed effect. Notably, this adjustment did not alter our main conclusions. Therefore, we present the results from mixed models, where nest ID served as a random effect. However in supplementary tables we also provide the results of analyses with nest ID considered as fixed effect.

For each initial model corresponding to each response variable, we selectively considered a subset of fixed effects likely associated with the response variable (Table 1 for details on expected relationships and mechanisms). Our approach involved commencing each analysis with an initial model, generating all possible simpler models, and presenting the top models ranked by the corrected Akaike information criterion (AICc) using the dredge function (Bartoń, 2013). In line with standard criteria and considering the paucity of data for model fitting, we considered fixed effects present in the top models within ΔAICc of 2.

Model validation involved using the simulateResiduals function from the “DHARMa” package (Hartig, 2022). This function was utilized for conducting various tests, including the Kolmogorov–Smirnov test for assessing uniform distribution, a dispersion test for detecting over/underdispersion, an outlier test, and the Levene test to evaluate homogeneity of variance. Multicollinearity among fixed effects was assessed using the variance inflation factor (VIF) function from the “car” package (Fox & Weisberg, 2019), with no significant issues observed. Both VIF and GVIF1/(2  × DF), where GVIF is the generalized VIF and DF is degrees of freedom, were lower than 1.15 (Fox & Monette, 1992; Kock & Lynn, 2012). The ggpredict function from the “ggeffects” package (Lüdecke, 2018) was employed to create figures illustrating the predicted values and confidence intervals. All statistics were conducted in R version 4.0.2 (R Core Team, 2021).

### Summary of parental provisioning and diet across the geographic range of the fairy pitta

We undertook a comprehensive review by compiling results of existing literature (Lin, Yao & Lee, 2007; Kim et al., 2012; Jiang et al., 2017, including our data) to determine whether earthworms constitute the primary component of the nestling diet across the pitta’s breeding range and to identify commonalities and differences in diet and provisioning behavior between southern and northern locations. We systematically tabulated (Table 2) the following information from all four studied sites (15 nests; including our data): general information (*e.g.*, clutch and brood size), nestling provisioning (*e.g.*, the number of prey items per visit), prey length, as well as diet composition (*e.g.*, the average proportion of earthworm).

**Table 2:** Summary of quantitative data on parental provisioning and nestling diet of the fairy pitta. The table includes information from southern locations (Lin, Yao & Lee, 2007; Jiang et al., 2017) and northern locations (Kim et al., 2012; this article). YES or NO or na (data not available to evaluate the statement).

Variables measured in a field study	Southern locations		Northern locations		Summary
	Taiwan^*^	South China	Jeju Island	Namhae-gun	
**GENERAL INFORMATION:**					
*1)* Breeding habitat	Bamboo plantations and secondary broadleaf forests	Limestone forests	Subtropical evergreen forests	Evergreen forests	Primary, secondary broadleaved forests and bamboo plantations.
*2)* Number of nests with diet studied/no. of food items (for Taiwan no. of items is not specified)	8/>1062^*^	1/354	2/826	4/647	15 nests/>2800 items from 4 study locations
*3)* Clutch size (range)/brood size (range)	3–5/3–5	5–6/4–5	?/?	5–6/5–6	3–6/3–6
**FEEDING TRIPS TO PROVISION NESTLINGS:**					
*4)* Number of visits per hour (range, mean ± SD)	2.2–7, ? ± ?	*na*, 3.9 ± 1.5	*na*, 2.8^*^ ± ?	*na*	Range: 2.2–7 per hour Mean: 2.8–3.9 per hour
*5)* Inter-visit interval (mean ± SD), minute	*na*	19 ± 11	*na*	35 ± 26	Mean: 19–35
*5)* Decrease in inter-visit interval with nestlings’ age	YES	YES	*na*	YES	YES
*6)* Effect of time of day on inter-visit interval: shortest in the morning	*na*	*na*	YES	YES	YES
*7)* Number of prey items per visit (mode, mean ± SD)	1, 1.8 ± 0.39	2, 2.05 ± 0.87	3, 3.0 ± 1.38	2, 2.74 ± 1.30	Mode: 1–3 per visit Mean: 1.8–3.0 per visit
*8)* Number of prey items higher in loads with earthworms	YES	*na*	YES	YES	YES
*9)* Parents sunder earthworms before carrying them to the nest	*na*	*na*	*na*	YES	YES
**DIET COMPOSITION:**					
*10)* If prevalence of earthworms	YES	YES	YES	YES	YES
*11)* Average % earthworms	73^*^	91.2	81.7	86	Typically, 80%–90%
*12)* Second/third/fourth most common prey taxon	caterpillars/ beetles/ vertebrates	Mantodea/ caterpillars/ Orthoptera	Homoptera larvae/?/?^&^	Centipedes/ Orthopterida/ beetles	Varies by location
*13)* Occasional presence of vertebrates in diet	YES	NO	NO	YES	Varies by location
*14)* Effect of rain on earthworms in the diet	YES, Between years	*na*	*na*	YES, Within a breeding season	YES
*15)* Decrease of % earthworms for older nestlings	YES^*^	*na*	*na*	YES	YES
*16)* Larger % of visits with earthworms for younger nestlings	YES^*^	*na*	*na*	YES	YES
**PREY SIZE:**					
*17)* Length of prey items (mode, mean ± SD), cm	4, 6.4 ± 0.55	*na*	*na*, 5.7 ± 2.85	5, 5.92 ± 2.68	Mode: 4–5 Mean: 5.7–6.4
*18)* Length of earthworms (most common size class, mean ± SD), cm	*na*	*na*	Size class: 5–6, 6.8 ± 2.39	Size class: 4–6, 6.34 ± 2.50	Most common: 4–6 Mean: 6.3–6.8

**Notes.**

* Estimate indirectly from data on the number of prey items per visit and per hour. The percentage appears to represent the % of visits in which an item was present, rather than the actual percentage among all food items, although the caption of Table 2 in Lin, Yao & Lee (2007) suggests differently. Additionally, the total number of items is not specified in the paper.

& Data from 2 nests pooled; % for only 2 taxa given.

### Estimation of brood earthworm consumption

We aimed to estimate the number and biomass of earthworms consumed by a brood during the parental provisioning period using our observational data. For each nest, we first summed the recorded number and biomass of earthworms for each day. These totals were then divided by the number of hours recorded on each particular day to obtain the number/biomass of earthworms per hour for each day. Next, we averaged the values to obtain the mean number/biomass of earthworm per hour across all days. We multiplied the mean values by the typical daily provisioning hours by pitta parents (15 h, from 5:00 to 20:00 h; based on JP’s observations) and further multiplied them by the duration of the nestling stage (12.05 days; mean value calculated from our data) to obtain the estimated number/biomass of earthworms provisioned during a breeding event (Table S3). We further divided the results by the brood size to obtain the estimated number/biomass of earthworms provisioned per nestling during a breeding event.

Finally, we calculated the mean estimated number/biomass of earthworms provisioned per nestling during a breeding event across all four nests (Table S3). We also calculated the mean value for three nests with more reliable data (excluding nest 2 with the smallest number of days with recordings; Tables S1 and S3). We multiplied the latter value by brood size of five to calculate the estimated number/biomass of earthworms consumed by a brood. The clutch size of the fairy pitta ranges from three to six, with three being rare (this study; Lin, Yao & Lee, 2007; Kim et al., 2012; Jiang et al., 2017). Therefore, we used a brood size of five in our calculations. We labeled the estimate as brood earthworm consumption.

### Estimation of parent earthworm consumption for calculating family earthworm consumption

In addition to brood earthworm consumption, we estimated parent earthworm consumption in order to estimate earthworm consumption for the entire family. First, we calculated the predicted number of earthworms consumed to fulfill the presumptive daily energy expenditure (DEE) of parents during a breeding event. The average mass of adult male and female fairy pittas is 109 g and 71.5 g, respectively (Erritzoe & Erritzoe, 1998). Based on these sex-specific body masses, we calculated the DEE of male and female using the following equation:

(1) \begin{eqnarray*}DEE=1092\times B{M}^{0.729},\end{eqnarray*}

where BM stands for body mass, and DEE is expressed in kJ, as derived by Kersten & Piersma (1987) for wading birds of a similar size to the fairy pitta. According to this equation, a pair with an average body mass has an estimated DEE of approximately 377 kJ per day. This equation has been previously applied in similar calculations by Onrust (2017) and is consistent with the correlation between body weight and DEE reported by Goldstein (1988) (see figure 6 there). It is similar to evaluations of DEE in species of similar size, such as the starling (*Sturnus vulgaris*; Daan, Masman & Groenewold, 1990) or the dipper (*Cinclus cinclus*; Bryant & Tatner, 1988; Williams & Vézina, 2001).

To express the estimated DEE in terms of earthworm biomass, we assumed that the energy content of earthworms collected by the fairy pitta is 16.72 kJ/g (AFDM) as measured by Yin, Xin & Qi (2007) in forest habitats of China. Finally, to convert this value into the number of earthworms, we assumed that an average earthworm weighs 0.12258 g AFDM, as indicated by our data.

Considering the lack of quantitative information on the proportion of earthworms in the parental diet, we considered that parents may have a much more diverse diet than nestlings (BirdLife International, 2001; Erritzoe, 2020). Moreover, earthworms do not constitute the majority of their diet. Therefore, we conducted calculations for five different values representing earthworm contributions to adult DEE: 30, 40, 50, 60, or 70%.

To account for parent DEE during the nest building and incubation periods, we considered that incubation lasts two weeks (based on our data) and the nest building stage lasts ten days (Kim, 1964 cited in Kim, 2014; Article S1). Finally, we used the following equation to estimate parent earthworm consumption during the breeding event (however, we acknowledge that our approximate equation does not account for potential variations in energy requirements across stages such as nest building, egg production, and incubation, as there is no data considering these aspects in the fairy pitta):

Estimated number of earthworms consumed by a parent during a breeding event = {proportion of earthworms in the adult DEE (0.3–0.7) × DEE for a pair (377 kJ/day) × number of days (12.05 + 14 + 10 days)}/{0.12258 g (average earthworm weight: AFDM) ×16.72 (kJ/g, AFDM of earthworms)}

The range of “0.3–07” indicates that the calculation was separately conducted for 30, 40, 50, 60, and 70% of earthworms in the adult DEE.

By adding this number to brood earthworm consumption, we obtained estimates of family earthworm consumption for each of the six proportions of earthworms in adult DEE (30, 40, 50, 60, or 70%).

### Estimation of home range size based on the earthworm consumption (“predicted home range”)

We aimed to determine the breeding home range size that is needed to meet the estimated brood and family earthworm consumption.

Earthworms are conventionally categorized into three functional groups (Lavelle et al., 2000): (1) epigeic, (2) endogeic, and (3) anecic. Epigeic earthworms, primarily found in the leaf litter (layer A0) of the forest floor, are the main group available to foraging pittas. Hence, we assumed that earthworm densities in the litter follow reported values for fairy pitta breeding habitats, ranging from 0.53 to 8.7 earthworms/m^2^ (Minamiya, Ishizuka & Tsukamoto, 2007; Kim et al., 2014).

In our calculations, we did not account for the impact of foraging pittas on earthworm abundance during the breeding period. Instead, we assumed balanced reproduction and mortality with minimal predation effects, resulting in a stable density of epigeic earthworms and their consistent surfacing frequency. This assumption is suitable for our calculations because macro-predators generally have weak effects on earthworm abundance (Kruuk, 1978; Macdonald, 1983; Judas, 1989; Schaefer, 1990).

We estimated home ranges as a function of the percentage (0–100%; equivalent to a proportion from 0 to 1) of earthworm abundance considered “available” to the birds. In this context, 0% implies that pittas cannot detect any earthworms, while 100% means that pittas can detect and capture all earthworms. To simplify our results, figures display only the results of these calculations for a narrower range (0–10%) of proportions of earthworms available to birds (see Methods ‘Assessing predicted and observed home range overlap’ section).

With these values and assumptions, we estimated the foraging home range size that would contain a number of surfacing earthworms for nestlings during the parental provisioning (brood earthworm consumption), and for a family during a full breeding event (family earthworm consumption) using the following equations:

home range size for brood [m^2^] = no. of brood earthworm consumption/{earthworm abundance (0.53 or 8.7 inds/m^2^) × proportion of surfacing earthworms (0.001–1)}

home range size for family [m^2^] = no. of family earthworm consumption/{earthworm abundance (0.53 or 8.7 inds/m^2^) × proportion of surfacing earthworms (0.001–1)}

### Literature-based estimations of breeding pair’s home range size/territory size (“observed home range”)

We aimed to compare the predicted home range size (Methods ‘Estimation of home range size based on the earthworm consumption’ section) with the area used by a breeding pitta pair. While there is no direct information on territory size or home range during nestling provisioning, fairy pittas establish territories through vocal advertisement and aggressive behavior. These behaviors result in spacing between breeding pairs that persists throughout the breeding season. Songbird parents extend their foraging range beyond their defended territory, potentially covering a larger area than their song-advertised territories (*e.g.*, Anich, Benson & Bednarz, 2009). Assuming a similar situation, we used the term “breeding home range” to focus on foraging rather than territorial defense.

We estimated the pitta’s breeding home range size using existing field observations, referred to as the “observed home range”. We used three types of literature sources for these estimates: behavioral observations of pitta pairs, density estimates in habitats harboring pitta populations, and a review of literature on territory or home range sizes in other similar-sized insectivorous bird species. A concise summary is presented here, with further details in Article S1.

**(1)** **Observations by Okada** (1999 cited in Kim, 2010; Article S1): Okada’s observations indicate that breeding birds are mainly found within 100–400 m from their nest, implying a circular home range size range of 3–50 ha. Using a midrange radius of 250 m, the hypothetical circular home range would be approximately 20 ha.

**(2)** **Transect-based estimates:** We used data from relatively non-fragmented Chinese forests (Jiang et al., 2017) to estimate home range size for each transect. The method relies on territorial vocalizations detected within a 200 m band in response to playbacks of the fairy pitta’s call. Considering varying responsiveness to vocalizations (see Article S1 for details), home range size estimates ranged from 22.6 to 45.3 ha. We suspect overestimation based only on density and believe the estimate of ∼20 ha is closer to reality, aligning with estimates from bird foraging behavior (no. 1 above).

**(3)** **Comparison with other species**: We examined literature on breeding territory or home range sizes in other insectivorous bird species, many of which were smaller in body size than the fairy pitta. These estimates varied from 6 to 35 hectares for birds of similar body size to the fairy pitta (see Article S1 for details).

Based on these approaches and considering that smaller body sizes in other species might have contributed to smaller territories/home ranges, we used a range of 10–30 ha for the “observed home range size” in our calculations. Conclusions remained consistent with ranges of 5–20 ha or 10–20 ha, with minor numerical differences in the degree of overlap (see Methods ‘Assessing predicted and observed home range overlap’ section) between observed and predicted home range sizes.

### Assessing predicted and observed home range overlap

We assessed the degree of overlap between predicted and observed home range sizes, considering the putative ability of pittas to detect epigeic earthworms. While there is no empirical data on pitta’s earthworm detectability, indirect evidence suggests that it is a small fraction of the total epigeic earthworm population. This inference is drawn from observations of seven epigeic earthworms measured by a human using a headlamp at night, revealing a range of 0–2.6% (average: 1.4%; median: 1.3%; calculations derived from data in Table 2 of Duriez, Ferrand & Binet, 2006). Pittas are likely more efficient than humans at night (Duriez, Ferrand & Binet, 2006) when visually searching for earthworms in shadowy places within the undergrowth during daytime. But virtually no quanititative data exist on this issue. For the purposes of this analysis, we estimated that pittas could exploit a range of 0.5–5% of the total number of epigeic earthworms; further research would be needed to determine this range more precisely.

In MATLAB (R2022a), we calculated overlap by integrating the area within the assumed earthworm availability domain (0.5–5%) at a resolution of 0.001%. We measured overlap for each predicted home range size under various conditions, including home range size for brood or family, and the percentage of earthworms in adults’ DEE (30 to 70%). The relative overlap index (%) was then determined by comparing calculated overlaps with the theoretically maximum overlap, representing the condition that results in the highest overlap. A higher relative overlap index value indicates that the chosen conditions in the calculations results in an estimate that more accurately reflects the observed home range size (10–30 ha).

### Literature-based summary of earthworm abundance

To discuss the conservation implications associated with the fairy pitta’s dietary specialization on earthworms, we conducted a literature-based summary of earthworm abundance in the fairy pitta’s breeding and wintering habitats. Moreover, to assess the possibility that anthropogenic disturbance might impact pitta populations, we expanded our investigation beyond natural habitats to include non-natural habitats, such as plantations, cultivated forests, and tree/shrub stands. While human activities in these areas can adversely affect nestling survival and reproductive success (Steidl & Anthony, 2000), these non-natural habitats may offer earthworms and vegetation cover, potentially serving as foraging grounds for the fairy pitta.

We present the data graphically to discuss conservation implications. But methodological variations, such as differences in soil depth sampled (ranging from 10 to 50 cm), render the dataset heterogeneous, preventing a statistical comparison of earthworm densities across habitat types. Since some studies hint at a positive correlation between the abundance of epigeic earthworms and all other types of earthworms (Fragoso & Lavelle, 1992; Paoletti, 1999; Hairiah et al., 2006), we compare total earthworm densities between natural and anthropogenic habitats, as well as between breeding and non-breeding grounds of the fairy pitta.

## Results

### Diet composition and prey length

We recorded 133, 49, 309, and 156 prey items in nests 1, 2, 3, and 4, respectively (Table S2) over 148 h of observations (details in Table S1). Among all 647 prey items, earthworms made up the largest proportion of the nestling diet (*n* = 547, 84.5%; Fig. 1C; Table S2; Video S1). Centipedes were the second most frequent prey item (*n* = 34, 5.3%). Other prey categories such as caterpillars, adult beetles, insect pupae, dragonfly, arrowhead flatworms, mole crickets, moth, grasshoppers, stick insects, and snake, comprised less than 9% of all prey items (details in Table S2).

**Figure 2:**
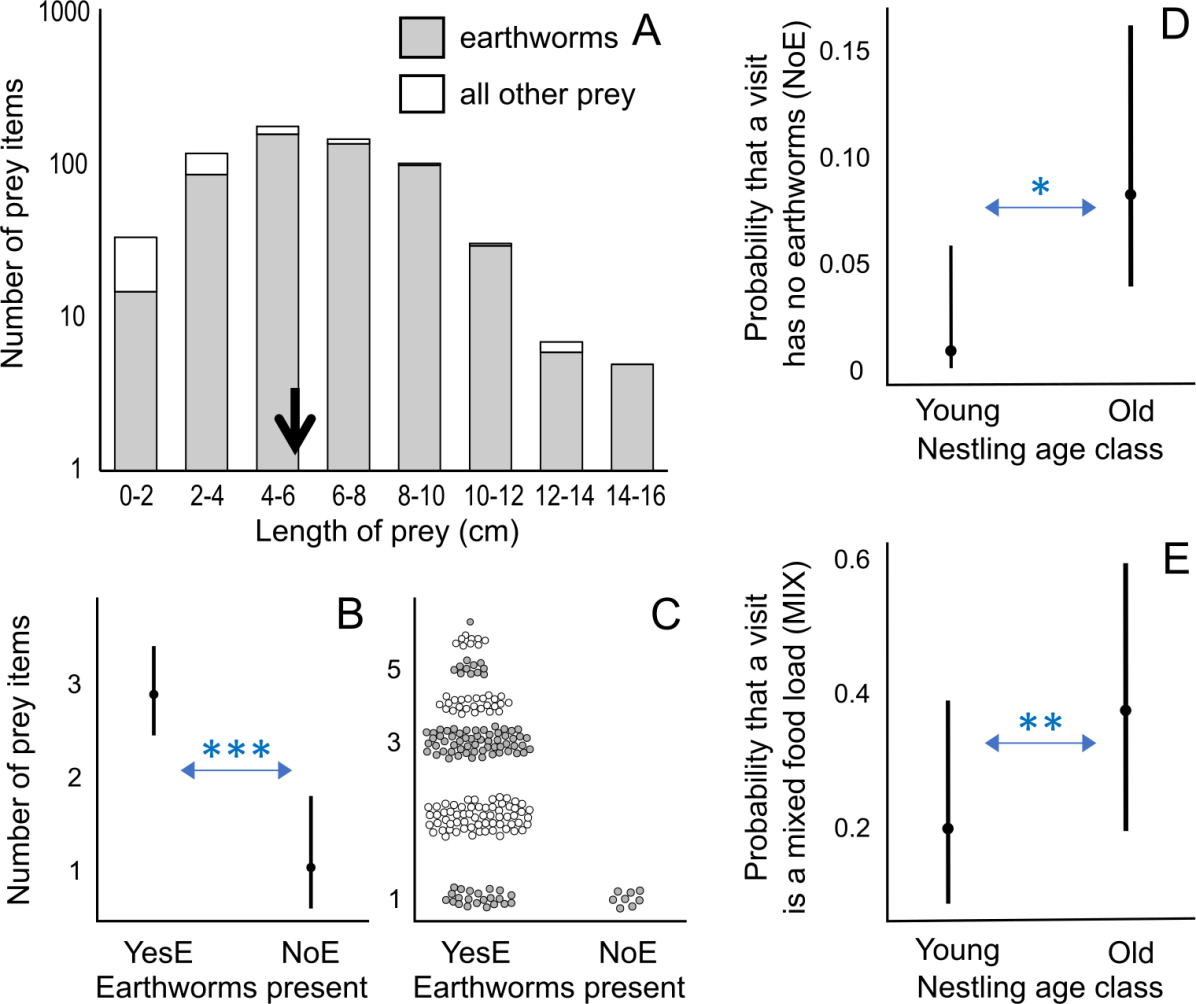
Prey length distribution and factors affecting number of prey items and visit types. (A) Distribution of prey length for all measured prey (*n* = 634), including first visits during observation sessions; the average length is indicated by a black arrow: 5.92 cm. (B) Effect of the presence of earthworms (YesE: earthworms present, *n* = 192; NoE: earthworms absent, *n* = 8) on the number of prey items per visit according to the lowest AICc model (Analysis 1 in Table 1; Table S4). The Conway–Maxwell–Poisson family with the log link function was applied due to a count response variable. (C) Raw data points (jittered for visualization) of the number of prey items per visit for two visit types (YesE, NoE) submitted to Analysis 1 (Table 1). (D) Effect of nestling age class on the probability (range: 0–1) of a feeding visit (*n* = 200 visits, excluding first visits per observation session; Analysis 3 in Table 1) lacking earthworms (“NoE”; statistical results in Table S6). (E) Effect of nestling age class on the probability (range: 0–1) that the visit containing earthworms (*n* = 192 “YesE” visits) featured the mixed food-load of earthworm and other prey (“MIX” visit type) according to the lowest AICc model (Analysis 3 in Table 1; Table S6). Additional results (Table S7) using age as a 12-level ordinal variable are in Fig. S1. For D and E, the Binomial family with the logit link function was used for binary response variables. In B, D, and E, the filled circles indicate predicted probabilities, and vertical bars represent 95% confidence intervals. For A and C, the *y*-axis is log-scaled for visualization. * indicates *p* < 0.05; ** indicates *p* < 0.01; *** indicates *p* < 0.001.

A total of 634 prey items were measured. We did not measure 13 earthworms (distributed as 7, 1, 2, and 3 among nests 1, 2, 3, and 4 respectively) observed in poor light conditions. The length of prey items varied from 0.49 to 15.49 cm (mean ± SD = 5.92 ± 2.68 cm, *n* = 634; Fig. 2A). Earthworms were relatively longer (6.34 ± 2.50 cm) than most of the other prey items (3.59 ± 2.40 cm), except for a small proportion (6.6%) of other prey items, including snake and arrowhead flatworms, which exceeded 10 cm. The longest prey was a 15.5 cm long earthworm.

The results of this study, along with literature (Table 2; Lin, Yao & Lee, 2007; Kim et al., 2012; Jiang et al., 2017), show that earthworms constitute the primary food for nestlings across their geographic breeding range. In addition to earthworms, the second most common prey in the nestling diet are caterpillars in the south and centipedes and cicada nymphs (“Homoptera larvae”) in the north (Table 2).

### Feeding visits and food-load content

Parents brought one to seven prey items per visit (mean ± SD = 2.74 ± 1.31, *n* = 233). The distribution of feeding visits across nests was as follows: 51 in nest 1, 18 in nest 2, 123 in nest 3, and 41 in nest 4, summing up to a total of 233 visits. Out of these visits, a mere 4.7% of all visits (11 out of 233) did not contain earthworms, while the remaining 95.3% (222 out of 233) included earthworms. Among the visits including earthworms, 64.8% (151 out of 233) exclusively comprised earthworms, while 30.5% (71 out of 233) represented a mixed food-load.

After excluding the initial visits in each observation session due to the inability to assess the preceding inter-visit interval, the sample sizes were as follows: 200 visits (all visits), 192 visits containing earthworms (“YesE” visits), 128 visits with only earthworms (“OnlyE” visits), and 8 visits without earthworms (“NoE” visits). These data served as the basis for LMER/GLMER analyses.

Visits with earthworms contained a higher number of prey items compared to visits without earthworms (earthworms present “NoE”: *β* ± SE = −1.031 ± 0.276, *p* < 0.001; Figs. 2B, 2C; Analysis 1 in Table 1; Table S4). Visits with earthworms consisted of 1 to 7 prey items, with a median of 3. Conversely, all statistically analyzed visits without earthworms consisted of one prey item (Fig. 2C). Additionally, during the initial visits (in each observation session), which were excluded from statistical analyses due to the unavailability of data on the preceding inter-visit interval, three food-loads lacking earthworms contained 2 prey items per load.

Among food-loads containing earthworms (*n* = 192), the mixed food-loads (range of 2–7 prey items, mean ± SD = 3.4 ± 1.2, median = 3) included more prey items (only earthworms “MIX”: *β* ± SE = 0.276 ± 0.059, *p* < 0.001; Analysis 1 in Table 1; Figs. S2A, S2B; Table S4) but fewer earthworms (only earthworms “MIX”: *β* ± SE = −0.174 ± 0.072, *p* < 0.05; Figs. S2C, S2D; Table S4) per load compared to visits exclusively containing earthworms (only earthworms “OnlyE”; range of 1–6 earthworms, mean ± SD = 2.6 ± 1.2, median = 2).

As the number of earthworms in food-loads with earthworms only (*n* = 128) increased, the average earthworm length per load decreased (*β* ± SE = −0.091 ± 0.028, *p* < 0.01; Fig. S3A; Table S5), and none of the other factors considered in this analysis (Analysis 2 in Table 1) were present in the top model. Bringing more earthworms (of the smaller size) per food load seemed to result in higher biomass of earthworms per load (Fig. S3B), but this positive correlation between the number and total biomass of earthworms per load disappeared after food loads with only one earthworm were excluded (Fig. S3B).

### Effect of nestling age and time of day

Older broods were more likely to receive food-loads without earthworms than younger nestlings (Fig. 2D; Analysis 3 in Table 1; Table S6), indicating that visits without earthworms occurred mostly when nestlings were older. When considering visits with earthworms, older nestlings were provisioned more frequently with mixed food-load visits than younger nestlings (Fig. 2E; Analysis 3 in Table 1; Table S6). The inter-visit interval and time of day were not included in the models with the lowest AICc value (Table S6). We also conducted an additional analysis coding age in 12 levels (nestlings of 1–12 days old; Table S7), where some levels were not represented in three out of the four nests (Fig. S1).

Classical contingency table analyses revealed a decrease in the proportion of earthworms among all prey items as nestling age increased (Fisher-combined test, *p* < 0.0001, Table S8). Utilizing the same method, we observed a decline in the frequency of earthworms-only feeding visits across all visit types as nestling age increased (Fisher-combined test, *p* = 0.001, Table S9). It is important to note that these relationships appeared to be driven by one nest (see Nest 1 in Tables S8 and S9). Therefore, caution should be exercised in generalizing these findings to the broader fairy pitta population.

The average inter-visit interval was 35 min (SD = ± 26, Min = 1, Q1 = 15, Q2 = 28, Q3 = 50, Max = 123, *n* = 200). Inter-visit intervals were shorter for old broods than for young broods (Figs. S4A, S4C; “young” *β* ± SE = 0.901 ± 0.325, *p*  < 0.01; Analysis 4 in Table 1; Table S10) and shorter in the morning than during the rest of the day (Figs. S4B, S4D; *β* ± SE = −2.149 ± 0.871, *p* < 0.05; Analysis 4 in Table 1; Table S10).

Finally, the number of earthworms in a load, biomass of a single earthworm, and the total biomass of earthworms per load did not differ between younger and older nestlings (Table S11). However, when considering nest ID as a fixed factor in linear models, the top model suggested that total biomass of all earthworms per visit is larger in younger broods (“young” *β* ± SE = 0.066 ± 0.044; Analysis 7 in Table 1; Table S11). This suggests a particular importance of earthworms for young nestlings.

### Effect of rain

**Figure 3:**
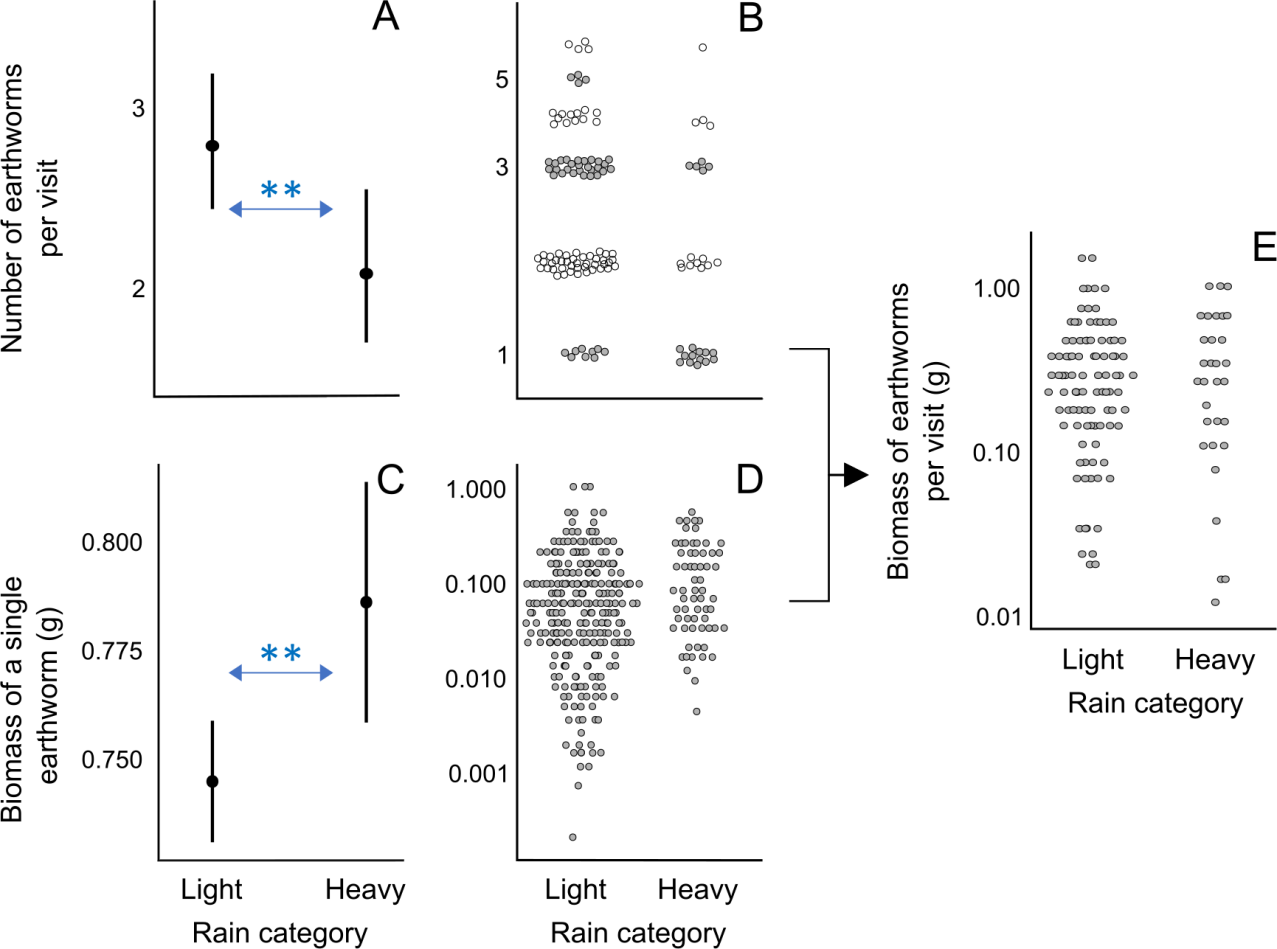
Effect of rain on the earthworm number and biomass in earthworm-only loads. (A) Effect of the rainfall category on the number of earthworms per visit according to the lowest AICc model (Analysis 5 in Table 1, Table S11). As the response variable is a count, we used the Conway–Maxwell–Poisson family with a log link function. (B) Raw data points of the number of earthworms per visit for light and heavy rainy days submitted to Analysis 5 (Table 1). (C) Effect of the rainfall category on the ash-free dry biomass of a single earthworm according to the lowest AICc model (Analysis 6 in Table 1; Table S11). The response variable was box-cox transformed (exponent value of 0.1) to improve the normality of model residuals. (D) Raw data points of biomass of a single earthworm for light and rainy days submitted to Analysis 6 (Table 1). (E) Raw data points of the ash-free dry biomass of earthworms per visit for light and rainy days submitted to Analysis 7 (Table 1), which resulted in no significant effects in the lowest AICc model (Table S11). The filled circles in A and C indicate predicted probabilities, with vertical bars indicating 95% confidence intervals. In B, D, and E, raw data points are jittered, and the *y*-axis is log-scaled for visualization. ** indicates *p* < 0.01.

In visits exclusively containing earthworms, the number of earthworms per visit was smaller on days with heavy rain compared to days with light rain (rainfall category “light rain”: *β* ± SE = 0.293 ± 0.099, *p* < 0.01; Figs. 3A, 3B; Analysis 5 in Table 1; Table S11). Although the model with the lowest AICc value for explaining the biomass of a single earthworm did not include the rainfall category, the second model with a nearly equivalent fit to the data, as indicted by ΔAICc = 0.33, included the effect of rain: earthworms brought to the nestlings were heavier on heavy rainy days compared to light rainy days (rainfall category “light rain”: *β* ± SE = −0.043 ± 0.014, *p* < 0.01; Figs. 3C and 3D; Analysis 6 in Table 1; Table S11). Rainfall category, as well as nestling age class and inter-visit interval, did not appear in the mixed model with ΔAICc < 2 explaining the total biomass of earthworms per visit (Fig. 3E; Analysis 7 in Table 1; Table S11). These results suggest that on heavy rainy days, parents deliver fewer but heavier earthworms, resulting in a similar total biomass of earthworms per food load compared to less rainy days (Fig. 3E).

Although the model with the lowest AICc does not incorporate the rain effect, its impact on inter-visit intervals in the three models within ΔAICc of 2 indicates that during days with heavy rain, pittas visited the nest at longer intervals (Table S10).

### Earthworm consumption and home range size

An average brood, from hatching to fledging, was estimated to consume approximately 985 earthworms (range: 619–1,359 earthworms; Table S3). This corresponds to approximately 699 g of fresh earthworm mass per brood in the observed nests (range: 398–1,009 g; Table S3). For a breeding event, we estimated that a pair of the fairy pittas and their brood consume 2,844–5,497 earthworms (Table S12), including 855 earthworms for five nestlings (Table S12). These estimated values depend on the assumed proportion of earthworms in parental DEE (30–70%; Table S12).

**Figure 4:**
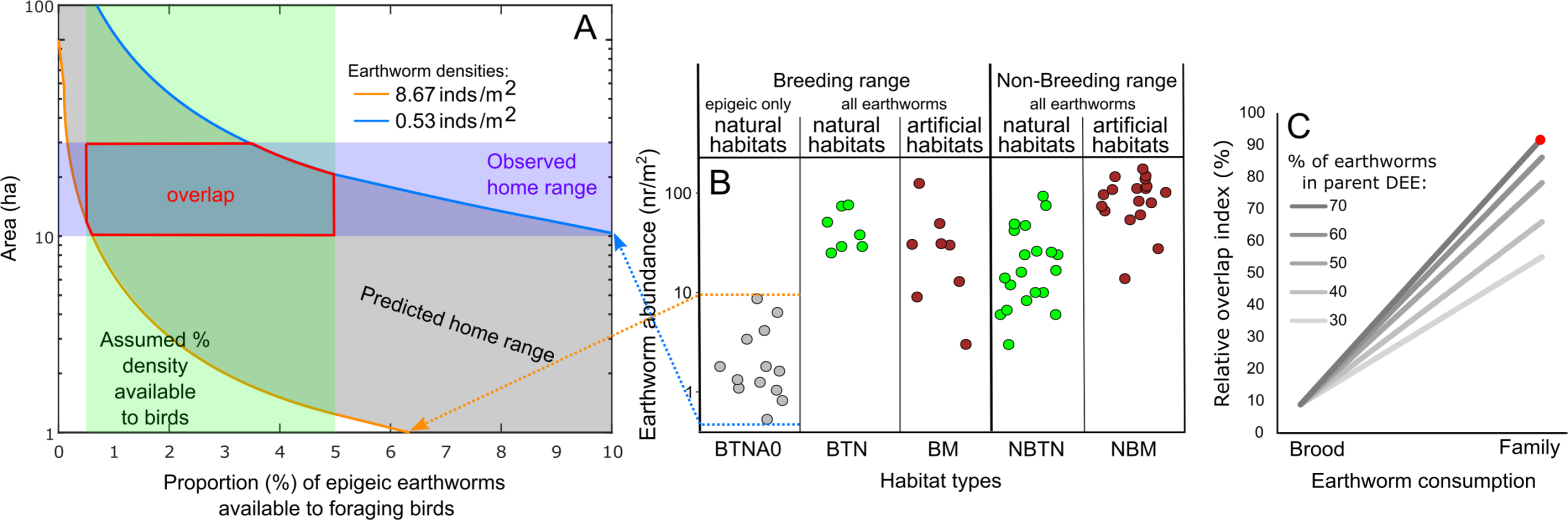
Relationships between earthworm densities, earthworm availability, predicted home range size for brood or family earthworm consumption, and observed home range size. (A) Example of the estimated range (gray diagonal band) of the area (ha, logarithmic scaled) that would contain the number of earthworms for family earthworm consumption (in this case 5,497 earthworms for a family with five nestlings, assuming that earthworms constitute 70% of the adult daily energy expenditure (DEE) as a function of earthworm availability (horizontal axis; percentage 0–10% shown). This assumes that epigeic earthworm densities vary between the minimum (blue line) and maximum (orange line) values detected in breeding habitats (Minamiya, Ishizuka & Tsukamoto, 2007; Kim et al., 2014). The vertical green band indicates the assumed range of values (0.5–5%) on the horizontal axis based on Duriez, Ferrand & Binet (2006). The horizontal purple band indicates the estimated range of the breeding home range size of the fairy pitta (10–30 ha). The section outlined in red indicates the overlap region between the predicted and observed home range sizes. This panel is taken from Fig. S5 (panel marked with an asterisk). (B) Earthworm densities (no. of individuals/m^2^, logarithmic-scaled; see Fig. S7 for non-transformed *y*-axis) from literature in different habitats: BTNA0–Breeding range, in Typical Natural habitats, soil layer A0 only; BTN–Breeding range, in Typical Natural habitats; BM–Breeding range, in Modified habitats that may provide sufficient vegetation cover for pittas to safely forage there; NBTN–Non-Breeding range, in Typical Natural habitats; NBM–Non-Breeding range, in Modified habitats that may provide vegetation cover for pittas to safely forage. Except for BTNA0, sampling was conducted from the soil surface to a variable depth deeper than the A0 layer. Biomass estimates are shown in Fig. S7. (C) Comparisons of the relative overlap between predicted and observed home range sizes in the given earthworm availability of 0.5–5% index for the brood and family earthworm consumptions. *Y*-axis indicates relative overlap index (%) with 100% indicating a theoretical maximum overlap. Five lines represent different earthworm percentages in parent DEE (30–70%). Red dot corresponds to the red polygon in A. Note that the *y*-axis in A uses a logarithmic scale for visualization, which was not applied in calculations of the relative overlap index in C.

Using these estimates, we calculated the home range size (gray diagonal band in Fig. 4A and Fig. S5) that would contain the number of earthworms fulfilling the brood and family earthworm consumption. We considered a range of epigeic earthworm densities found in pitta habitats (Fig. 4B), from the lowest (blue line in Fig. 4A) to the highest (orange line in Fig. 4A). Moreover, we took into account a range of earthworm availability to parents (*x*-axis in Figs. 4A and S5).

We calculated the degree of overlap (red polygon in Figs. 4A and S5) between the predicted home range size values (gray band) and observed home range size (violet horizontal band) within the considered range of earthworm availability proportions (green vertical band). The extent was lower when considering only brood consumption compared to including parental consumption (Fig. 4C). Moreover, the degree of overlap was the greatest when assuming that earthworms compose a relatively substantial proportion (70%) of parental DEE (Fig. 4C). Even with alternative home range sizes (5–20 ha or 10–20 ha), we observed a 99% relative overlap index when assuming earthworms compose 60% or 70% of parental DEE during the breeding event (Fig. S6). Notably, a relatve overlap index exceeding 90% was observed for all three home range size options only under the assumption of earthworms accounting for 70% of parental DEE (Figs. 4C and S6).

### Abundance of earthworms in different habitats

Information regarding epigeic earthworm densities in pitta habitats is scarce, and the available estimates are shown in the leftmost column of Fig. 4B. Earthworm biomass data, on the other hand, is too scarce to draw reliable conclusions (Fig. S7). Within the geographic breeding range of the fairy pitta, earthworm densities were generally lower in anthropogenic habitats (*e.g.*, plantations) than in natural breeding habitats (Fig. 4B). On the wintering grounds (non-breeding range in Fig. 4B), several anthropogenic habitats (Somniyam & Suwanwaree, 2009; Chakraborty & Chaudhuri, 2017; Darmawan et al., 2017) exhibit a relatively higher abundance/biomass of earthworms compared to natural habitats (Figs. 4B, S7; see also Data S1).

## Discussion

### Nestling diet and parental provisioning

The results of this study, combined with existing literature on parental provisioning and the nestling diet of the fairy pitta, confirm that earthworms are the primary food for nestlings across their geographic breeding range. While earthworms dominate the nestling diet throughout the fairy pitta’s breeding range, the prey types comprising the rest of their diet vary by region. The next most common prey type is caterpillars in the south, and centipedes and cicada nymphs (“Homoptera larvae”) in the north. As earthworms were, on average, larger than other prey items, and the number of prey items per visit was larger when the prey load included earthworms, the results indicate the importance of earthworms for providing nestlings with food loads of high nutritional value that are easy to swallow because they do not contain hard exoskeletons. The high number of prey in earthworm-containing loads may suggest a preference for foraging on earthworms or a high abundance of earthworms in the habitat.

Among visits involving earthworms, a comparison between those exclusively featuring earthworms and those incorporating mixed food items shows that the number of earthworms is higher in visits with only earthworms, while the total number of prey items is higher in mixed food-loads. Assuming most visits comprise a full beak load (*e.g.*, Fig. 1A), this may suggest that when foraging pittas are unable to find enough earthworms, they resort to non-earthworm prey items of smaller size, resulting in higher number of items in a beak-load because non-earthworm types are not sundered (Park et al., 2022) and take less space in the beak. Alternatively, if birds aim to deliver a load of similar nutritional value on average, it implies one earthworm is replaced with several smaller, less nutritious prey items. Given our exclusive focus on direct empirical documentation of nestling diet rather than parental foraging behavior, future research focusing on parental behavior would be able to determine parental prey preferences relative to available prey, foraging decisions, prey energy content, and their effects on brood size and breeding success.

In earthworm-only food-loads containing multiple earthworms, the total biomass per load remained constant irrespective of the number of earthworms present because birds brought either a smaller number of larger earthworms or a larger number of smaller earthworms. This observation suggests that, when delivering food, birds tend to bring consistent biomass of earthworms per visit regardless of prey items’ size. Although we expected an increase in the biomass of a beak-load with the age of nestlings, no such positive correlation was found. The lack of a positive effect of nestling age on the number of earthworms in a load, the biomass of a single earthworm, or the total biomass of earthworms per load further suggest that birds tend to bring a consistent biomass delivery per visit regardless of nestling age. We hypothesize that this behavior may be attributed to the parents’ inclination to maximize the filling of their beaks, a phenomenon frequently observed in the field (*e.g.*, Fig. 1A). Loads consisting of only one earthworm deviated from this pattern by being lighter than loads with multiple earthworms.

The intervals between parental provisioning visits to nests are the shortest in the morning in both southern and northern regions, reflecting the typical provisioning pattern of passerine birds (Pinkowski, 1978; Johnson & Best, 1982; Knapton, 1984). Pittas in the north breeding range (our study; Kim et al., 2012) bring more prey items during each visit at longer inter-visit intervals compared to the south region (Lin, Yao & Lee, 2007; Jiang et al., 2017). This regional difference might be attributed to their habitat characteristics, such as the distance between the nest and available foraging patches, the number of foraging patches, and prey density in the foraging area, indicating that future research should focus on quantifying the distribution and abundance of pitta’s prey animals within their home ranges across different geographical locations

### Effect of rain

The prevalence of earthworms in the nestling diet of the fairy pitta is affected by precipitation patterns. Specifically, rain affects the abundance and surfacing behavior of earthworms (Edwards et al., 1995). Our data shows that the short-term effect of heavy rain resulted in delivery of fewer earthworms per visit, although those worms were longer. This result, coupled with the lack of effect of rain on the total biomass per load, suggests that, on average, parents tend to bring a full beak load at each visit, as already discussed above (in “Nestling diet and parental provisioning”). A possible explanation for the observed decline in numbers is that larger earthworms may become more available to birds during and immediately after rainy weather, when they are more likely to be observed on surface. Another possibility is that fewer large (*i.e.,* thick and long) earthworms can fit into the full-beak load of food, partly due to larger thickness and partly due to the sundering (Park et al., 2022) of longer earthworms into a larger number of pieces carried in the beak (Fig. 1A).

The larger size of worms collected on heavy rain days may be related to their surfacing behavior. We hypothesize that heavy rain may not only increase the number of earthworms but also elevate the proportion of large worms available to birds. This could lead to greater selectivity towards larger prey, as predicted from optimal foraging theory (Schoener, 1971; Charnov, 1976; Pyke, Pulliam & Charnov, 1977). Future studies should compare handling and search times for prey of different sizes and determine the effect of rain on earthworm availability in breeding habitats of the fairy pitta. This will further help to evaluate the effects of rain on foraging behaviors and brood provisioning.

It is also possible that foraging birds may cue on the sounds of earthworms moving in leaf litter (Montgomerie & Weatherhead, 1997). Consequently, during dry weather or light rain when the litter produces clear sounds from earthworm movements, the number of earthworms available to foraging pittas may be higher than the visually detectable proportion. These auditory cues are less likely to be beneficial when dead leaves are wet after heavy rain, but perhaps larger worms still make sounds, allowing pittas to detect them even in heavy rain, whereas smaller worms are too quiet. To fully understand the effect of rain on the foraging strategy, detailed observations on foraging adults are required.

### Earthworm consumption and home range size

Our estimates suggest that pitta nestlings consume between 619–1,359 earthworms during the parental provisioning period. These estimates are generally consistent with Okada’s estimate of 70–80 earthworms daily (Okada, 1999 cited in BirdLife International, 2001; Article S1), which would amount to approximately 910–1,040 earthworms during the parental provisioning period.

Based on estimated family earthworm consumption, we calculated the predicted breeding home range size that would provide a family with this number of earthworms. The highest degree of overlap between predicted and observed home range sizes occurred when we assumed that 70% of the parental daily energy expenditure (DEE) is derived from earthworms. This extrapolation provides indirect evidence that a large portion of the adult pittas’ diet, for which no quantitative data exist, consists of earthworms.

Our approach does not account for breeding pairs in fragmented landscapes, where only certain portions of their home range have habitat patches with varying foraging costs and access to earthworms. To comprehensively assess how pittas adjust to different conditions and optimize their territorial and foraging behavior, more direct foraging observations are essential. Future research should involve precise assessments of home range size, food availability, habitat patchiness, foraging costs, and other relevant factors. These details, particularly the adult diet and the evaluation of earthworm detectability, will enhance the accuracy of our home range modeling and provide valuable insights relevant to conservation.

### Implications for conservation

The population sizes of dietary specialists are closely linked to the abundance of their preferred food items (Martay & Pearce-Higgins, 2020). Our results directly show that earthworms are the primary food source for fairy pitta nestlings and indirectly support the view that they are also the primary food of their parents during the breeding period. This evidence-based connection between the pitta family’s diet and their reliance on earthworms underscores the important role of earthworm-focused strategies in effective conservation efforts.

**Figure 5:**
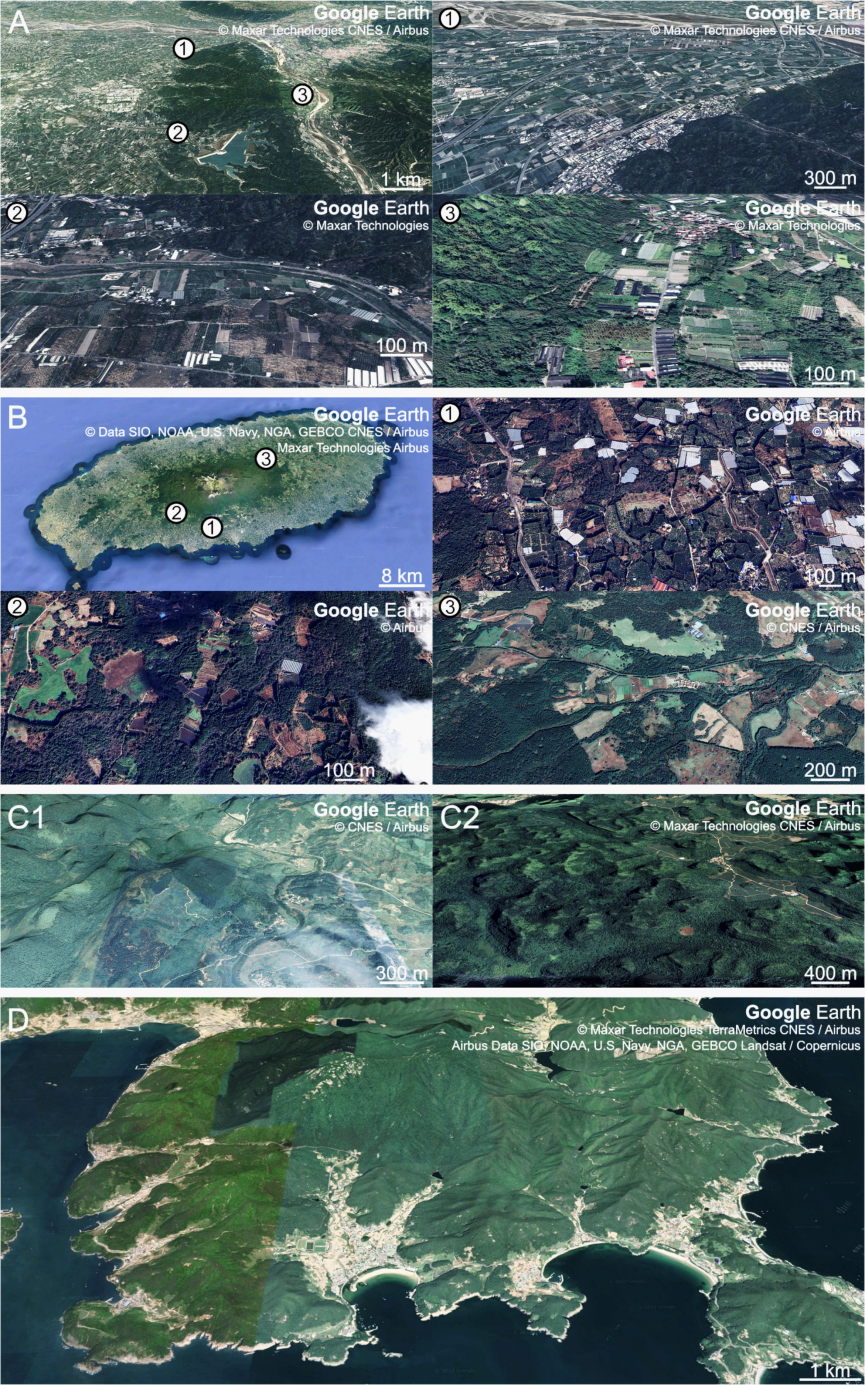
Aerial views of breeding habitats of the fairy pitta with different degrees of fragmentation and surrounding agricultural landscapes. (A) Breeding habitat in Linnei (23°44.5′N, 120°37′E), Taiwan, study site of Lin, Yao & Lee (2007). Panels (nos. 1, 2, 3) provide enlarged views of marked points in the main figure. (B) Breeding habitat in Jeju (33°20′N, 126°31′E), South Korea, study site of Kim et al. (2012). Panels (nos. 1, 2, 3) show enlarged views of marked points in the main figure. (C1, C2) Breeding habitats in Mulun (C1; 25°08′N, 108°03′E) and Nonggang (C2; 22°28′N, 106°57′E), China, study sites of Jiang et al. (2017). (D) Breeding habitat in Namhae-gun (34°44′N, 127°59′E), South Korea, study site of this article. Map data Google Earth ©2022 Maxar Technologies CNES / Airbus for A, panel no. 1 in A, and C2; ©2022 Maxar Technologies for panels nos. 2 and 3 in A; ©2022 CNES / Airbus for C1 and panel no. 3 in B; ©2022 Data SIO, NOAA, U.S. Navy, NGA, GEBCO CNES / Airbus Maxar Technologies Airbus for B; ©2022 Airbus for panels nos. 1 and 2 in B; ©2022 Maxar Technologies TerraMetrics CNES/Airbus Airbus Data SIO, NOAA, U.S. Navy, NGA, GEBCO Landsat/Copernicus for D.

The fairy pitta prefers natural and undeveloped areas (Ko et al., 2009). However, the breeding regions are often occupied by plantations (Fig. 5), and habitat destruction is considered the primary driver of pitta population decline (BirdLife International, 2001; BirdLife International, 2017; BirdLife International, 2020). This habitat loss reduced the availability of earthworm-rich areas for pittas, which may lead to heightened competition among earthworm-consuming animals. Conservation efforts to support fairy pitta breeding events should focus on ensuring an ample supply of earthworms. This involves increasing earthworm availability and facilitating earthworm foraging in man-made habitats rich in earthworms located near natural habitats.

One approach could involve enhancing earthworm abundance in natural habitats by introducing ground vegetation cover to retain soil moisture (Barras, Candolfi & Ouzel, 2022). Additionally, the application of nitrogenous fertilizers (*e.g.*, ammonium sulfate; Edwards & Lofty, 1982; Yahyaabadi, Hamidian & Ashrafi, 2018) could be considered to increase the availability of earthworms for pittas. However, the use of fertilizers requires thorough assessment to mitigate potential environmental impacts.

Another approach stems from the observation that pittas use modified forest habitats (BirdLife International, 2020). Certain anthropogenic habitats within the breeding range exhibit earthworm abundance comparable to natural forests (*e.g.*, hedges in agricultural landscape, Fang et al., 1999; Fig. 4B). These man-made habitats present opportunities for enhancing conservation initiatives. Pitta parents frequently visit anthropogenic forests because of the high earthworm densities in those habitats (Fujida, Okeguchi & Sawada, 1992 cited in BirdLife International, 2001; Article S1). In Japan and Taiwan, fairy pittas establish breeding territories within or near plantations (Lin, Yao & Lee, 2007; Minamiya, Ishizuka & Tsukamoto, 2007), suggesting that certain anthropogenic alterations may be acceptable for breeding pittas by providing a rich abundance of crucial food resources. Consequently, we could consider providing covers, such as bushes and canopies, in anthropogenic areas abundant with earthworms near natural breeding habitats. This would facilitate safe foraging for pittas, offering easy access to essential food resources.

Our review of earthworm densities in pitta wintering grounds suggests that considering actions in non-breeding areas, particularly by incorporating anthropogenic habitats into plans, could enhance overall effectiveness. Several anthropogenic habitats on these wintering grounds, including managed secondary forest, mixed and homogenous plantations with bushes and vegetation cover, as well as bamboo plantations (Somniyam & Suwanwaree, 2009; Chakraborty & Chaudhuri, 2017; Darmawan et al., 2017), harbor a comparatively higher abundance/biomass of earthworms than natural habitats. We hypothesize that the high abundance of earthworms in anthropogenic habitats on wintering grounds may prompt pittas to forage in these areas. This experience could potentially extend to the breeding grounds, especially if anthropogenic habitats there contain a well-developed leaf litter layer, in which epigeic earthworms live.

When evaluating the aforementioned conservation approaches, it is crucial to consider the size and distribution of earthworm-rich habitat patches in fragmented landscapes. Pittas are unlikely to exploit food from distant natural habitat patches, and in Taiwan and Jeju, natural habitats appear to be isolated and intermixed (Figs. 5A, 5B) with plantation patches (*e.g.*, mandarin orange plantations, Fig. 5B). This isolation of natural habitat patches suggests a potential heightened competition for earthworms in these regions, demanding prioritized action. To address this, promoting anthropogenic habitats suitable for pittas’ foraging adjacent to the natural habitats and enhancing earthworm abundance in natural habitats could be effective. Meanwhile, natural habitats are fragmented by plantations in China (Fig. 5C), and they are surrounded by rice plantations in Namhae (Fig. 5D). However, these natural habitats are relatively extensive and can provide sufficient nesting sites for breeding territories and foraging home ranges. Therefore, conservation strategies in these regions could focus on increasing earthworm abundance specifically in natural habitats.

Finally, if the intentional exploitation and destruction of natural breeding habitats is unavoidable, conservation strategies may include replacing these natural habitats by creating a mosaic of both natural and anthropogenic habitat patches rich in earthworms. The determination of patch sizes should be guided by local earthworm density, with an ideal range of 10–30 ha aligning with the expected breeding home range of pittas. Certain types of plantations may indeed provide a relatively high earthworm abundance (as indicated in Fig. 4B), as well as cover for foraging birds. Additionally, based on our observations of feeding patterns, conservation efforts should advise against activities near nests in the morning, as this period corresponds to the time of the highest feeding rate.

We discussed here conservation strategies for one of the diet specialists, the fairy pitta, but these approaches may potentially be considered for the conservation of other vermivores dependent on earthworm-rich habitats, such as other pittas (Pittidae) antpittas (Grallaridae), thrushes (Turdidae), woodcocks (*Scolopax*), and lapwings (*Vanellus*; Reynolds, 1977; Onrust, 2017; Collar, 2020; Schulenberg & Kirwan, 2020; Winkler, Billerman & Lovette, 2020).

## Conclusions

Earthworms are the primary food type for fairy pitta nestlings, as supported by direct evidence, and their parents, as indicated by indirect evidence. Young nestlings’ diet is especially dominated by earthworms. The range of observed breeding home range sizes in natural habitats is consistent with the idea that the fairy pitta’s typical spacing behavior (territoriality, foraging home range) may be adjusted to encompass the areas of natural habitats that provide earthworms for family consumption during a full breeding event. However, quantifying correlations between local earthworm densities, home range size, and breeding success of individual pairs is needed. The results highlight the importance of earthworm-rich habitats in conservation planning. Conservation actions could include enhancing the earthworm abundance in natural habitats to mitigate habitat destruction (BirdLife International, 2001). Additionally, we could consider providing vegetation cover in adjacent earthworm-rich anthropogenic habitats, given previous reports of breeding in such plantations. Future studies should further examine whether habitat selection and breeding success are indeed linked to high earthworm abundance.

## Supplemental Information

10.7717/peerj.17189/supp-1 Supplemental Information 1 Effect of nestling age on feeding visit types (A) Effect of nestling age on the probability (range: 0–1) of visits that do not contain earthworms (earthworms present: NoE). (B) Effect of nestling age on the probability (range: 0–1) of visits that contain earthworms and other prey items (only earthworms: MIX). The panels are derived from the model with the lowest AICc value that explains the variation in the response variable (Table S7). For both, the response variable is a binary (coded as 0 or 1), we used a Binomial family with the logit link function. Details of statistical analyses are provided in Table S7. The numbers below each nestling age refer to the number of nests (out of a total of 4) contributing to the data used in the analyses. Curved line indicates predicted probabilities of NoE (A) or MIX (B) visit type, and gray-shaded area represents 95% confidence intervals. * indicates *p* ¡ 0.05; ** indicates *p* ¡ 0.01.

10.7717/peerj.17189/supp-2 Supplemental Information 2 Relationship between the earthworm-containing feeding visit types and number of prey items (A) Effect of visit types (only earthworms: OnlyE, or mixed food-load: MIX) among earthworm-containing food loads (YesE, *n* = 192) on the number of prey items per load (Analysis 1 in Table 1). (B) Raw data points of the number of prey items for visits with only earthworm and visits with mixed food-load submitted to analysis 1. (C) Effect of feeding visit type (only earthworms: only earthworms or mixed food-load) on the number of earthworms per load (Analysis 1 in Table 1). (D) Raw data points of the number of earthworms for visits with only earthworm and visits with mixed food-load submitted to analysis 1. A and C are derived from the model with the lowest AICc value that explains the variation in the response variable (Table S4). For both, the response variable is a count, and we used a Poisson family with the log link function. Details of statistical analyses are provided in Table S4. The filled circles indicate predicted probabilities, and vertical bars represent 95% confidence intervals. * indicates *p* ¡ 0.05; *** indicates *p* ¡ 0.001. In B and D, raw data points are jittered, and the *y*-axis is log-scaled for visualization.

10.7717/peerj.17189/supp-3 Supplemental Information 3 Relationships between earthworm number (*x*-axis) and the average earthworm length per visit (A) or the total biomass of food-load in earthworm-exclusive visits (B) (A) Effect of the number of earthworms per visit on average earthworm length, with predicted values (red line) and confidence intervals (shaded area) from the lowest AICc model explaining variation in the response variable (Analysis 2 in Table 1; Table S5). In the LMER model, the average earthworm length per visit (*y-* axis) was square-root transformed to improve the normality of model residuals. (B) Positive correlation (Pearson *r* = 0.203, n = 128 visits, *p* ¡ 0.05; blue solid line: *y* = 0.049*x* + 0.227, R^2^ = 0.041) exists between the number of earthworms per visit (*x*) and the biomass of earthworms per visit (*y*). This relationship illustrates the positive correlation between the two variables representing distinct aspects (number *vs.* biomass) of earthworm amount during a single visit. However, this correlation is caused by lower *y*-values associated with visits featuring only one earthworm (*x* = 1), and it loses significance for *x* values between 2 and 6 (Pearson *r* = 0.104, *n* = 106 visits, *p* = 0.287; black dashed line: *y* = 0.029*x* + 0.299, R^2^ = 0.011). Both *y*-axes are log-scaled for visualization.

10.7717/peerj.17189/supp-4 Supplemental Information 4 Factors affecting the inter-visit interval (A) Effect of nestling age class on the inter-visit interval. (B) Effect of time of day on the inter-visit interval. M, N, and A represent morning (8:00 to 10:00 h), noon (10:00 to 14:00 h), and afternoon (14:00 to 18:00 h), respectively. Panels A and B are derived from the model with the lowest AICc value that explains the variation in the response variable (Analysis 4 in Table 1; Table S10). In the statistical analysis, the response variable was square-root transformed to improve the normality of model residuals. Details of statistical analyses are shown in Table S10. The filled circles indicate predicted values of the inter-visit internal (in minutes), and vertical bars represent 95% confidence intervals. *n.s.* indicates non-significant; * indicates *p* ¡ 0.05; ** indicates *p* ¡ 0.01. (C) Raw data points of inter-visit intervals for young and old nestlings submitted to Analysis 4 (Table 1). (D) Raw data points of inter-visit intervals for morning, noon, and afternoon submitted to Analysis 4 (Table 1). In C and D, raw data points are jittered, and the *y*-axis is log-scaled for visualization.

10.7717/peerj.17189/supp-5 Supplemental Information 5 Relationships between earthworm densities, earthworm availability, predicted home range size for brood or family earthworm consumption, and observed home range size The gray band represents the estimated range of habitat area (vertical axis; ha, logarithmic scaled) that contains a number of earthworms to meet brood or family earthworm consumption. This estimation depends on the proportion (horizontal axis; percentage range 0–10% shown) of local earthworm density available to foraging pittas. The lower (orange) and upper (blue) edges of the gray band correspond to estimates calculated for the highest and lowest values, respectively, of epigeic earthworm densities in the fairy pitta habitats (Minamiya, Ishizuka & Tsukamoto, 2007; Kim et al., 2014). The vertical yellow band indicates the range (0.5–5%) on the horizontal axis likely to occur in nature based on Duriez, Ferrand & Binet (2006; see Methods ‘Assessing predicted and observed home range overlap’ section). The horizontal purple band indicates the estimated breeding home range size of the fairy pitta (10–30 ha). In each panel, the area marked with the red-shaded polygon indicates the ‘overlap’ region presenting predicted home range size that meets the observed home range size in the given earthworm availability (0.5–5%). The panels are arranged in two columns: brood or family earthworm consumption. The five rows represent different proportions of earthworms in the parent DEE. The panel marked with an asterisk is presented in Fig. 4A. The *y*-axis uses a logarithmic scale for visualization.

10.7717/peerj.17189/supp-6 Supplemental Information 6 Comparisons of the relative overlap index for alternative observed home range sizes of 10–20 or 5–20 ha The figures show the relative overlap index for brood and family earthworm consumption. The calculations were based on an observed home range size of either 10–20 ha (A) or 5–20 ha (B). *Y*-axis indicates relative overlap index (%) with 100% indicating a theoretical maximum overlap. The five lines represent situations that differ in the % of earthworms in the parent DEE ranging from 30% to 70%.

10.7717/peerj.17189/supp-7 Supplemental Information 7 Summary of literature on earthworm densities (A) and biomass (B) in natural habitats and in anthropogenic habitats, including various plantations Habitat types: **BTNA0** –Breeding range, in Typical Natural habitats, soil layer A0 only; **BTN** –Breeding range, in Typical Natural habitats; **BM** –Breeding range, in Modified habitats that may provide sufficient vegetation cover for pittas to safely forage there; **NBTN** –Non-Breeding range, in Typical Natural habitats; **NBM** –Non-Breeding range, in Modified habitats that may provide sufficient vegetation cover for pittas to safely forage there. Except for **BTNA0**, sampling was conducted from the soil surface to a variable depth deeper than the A0 layer. Data are tabularized in Data S1. (C, D) These are additional figures for A and B, using a logarithmic scale on the y-axes for visualization.

10.7717/peerj.17189/supp-8 Supplemental Information 8 Supplementary tables

10.7717/peerj.17189/supp-9 Supplemental Information 9 The fairy pitta feeding the nestlings A parent with a full load of earthworms arrives at the nest entrance and feeds the nestlings inside. Video credit: Jinseok Park.

10.7717/peerj.17189/supp-10 Supplemental Information 10 Supplementary Methods

10.7717/peerj.17189/supp-11 Supplemental Information 11 Earthworm abundance data The abundance of earthworms in different habitats and includes references.

10.7717/peerj.17189/supp-12 Supplemental Information 12 Nestling diet data The nestling diet of the fairy pitta.

## Additional Information and Declarations

### Data Availability

The following information was supplied regarding data availability: The raw data are available in the Supplemental Files.
