# Supplementary figures and images for: Assessing dietary specialization to inform the conservation of the fairy pitta (Pitta nympha), an endangered vermivore
Source: PeerJ. 2024 Apr 29;12:e17189. doi: 10.7717/peerj.17189 (PMC11064856; doi:10.7717/peerj.17189)

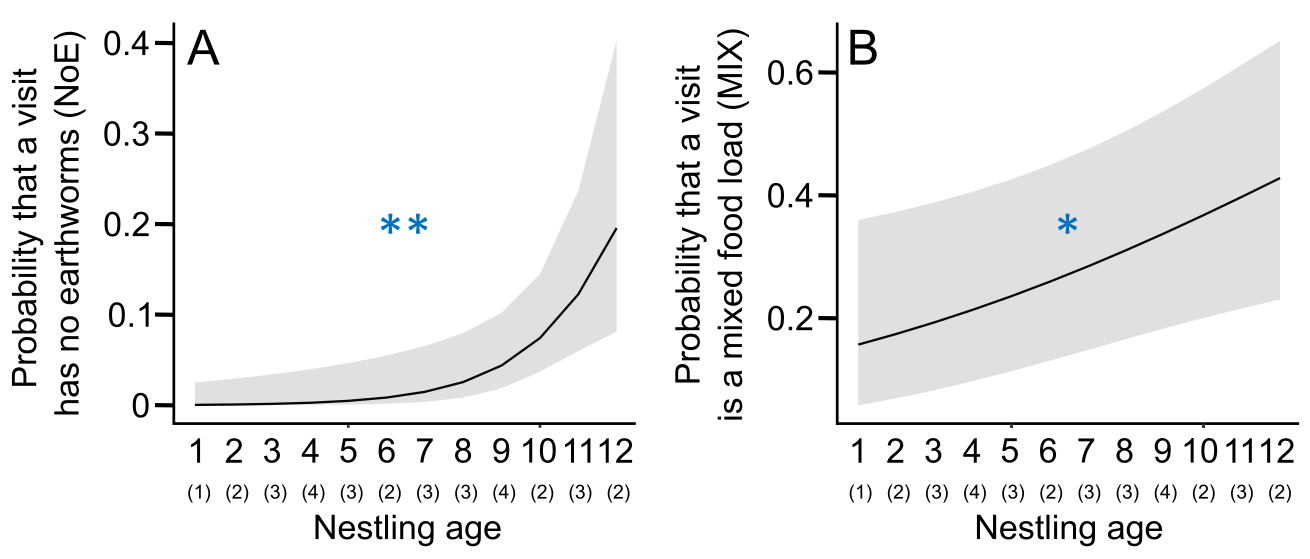

Supplement: Supplemental Information 1 — (A) Effect of nestling age on the probability (range: 0–1) of visits that do not contain earthworms (earthworms present: NoE). (B) Effect of nestling age on the probability (range: 0–1) of visits that contain earthworms and other prey items (only earthworms: MIX). The panels are derived from the model with the lowest AICc value that explains the variation in the response variable (Table S7). For both, the response variable is a binary (coded as 0 or 1), we used a Binomial family with the logit link function. Details of statistical analyses are provided in Table S7. The numbers below each nestling age refer to the number of nests (out of a total of 4) contributing to the data used in the analyses. Curved line indicates predicted probabilities of NoE (A) or MIX (B) visit type, and gray-shaded area represents 95% confidence intervals. * indicates p ¡ 0.05; ** indicates p ¡ 0.01. [file peerj-12-17189-s001.png]

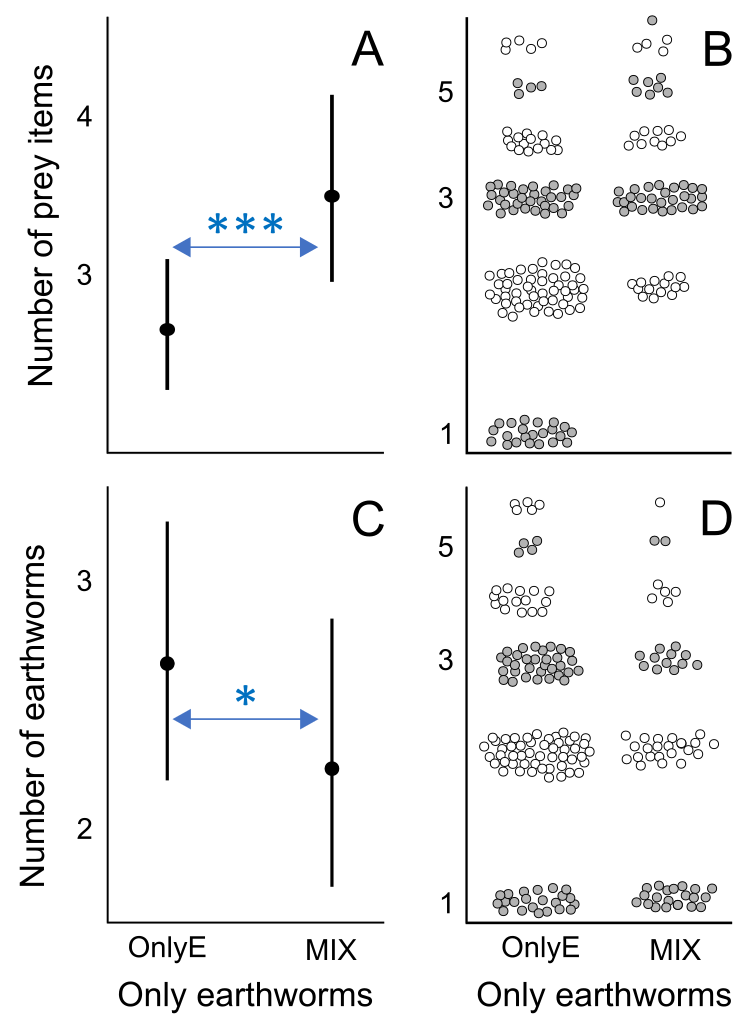

Supplement: Supplemental Information 2 — (A) Effect of visit types (only earthworms: OnlyE, or mixed food-load: MIX) among earthworm-containing food loads (YesE, n = 192) on the number of prey items per load (Analysis 1 in Table 1). (B) Raw data points of the number of prey items for visits with only earthworm and visits with mixed food-load submitted to analysis 1. (C) Effect of feeding visit type (only earthworms: only earthworms or mixed food-load) on the number of earthworms per load (Analysis 1 in Table 1). (D) Raw data points of the number of earthworms for visits with only earthworm and visits with mixed food-load submitted to analysis 1. A and C are derived from the model with the lowest AICc value that explains the variation in the response variable (Table S4). For both, the response variable is a count, and we used a Poisson family with the log link function. Details of statistical analyses are provided in Table S4. The filled circles indicate predicted probabilities, and vertical bars represent 95% confidence intervals. * indicates p ¡ 0.05; *** indicates p ¡ 0.001. In B and D, raw data points are jittered, and the y-axis is log-scaled for visualization. [file peerj-12-17189-s002.png]

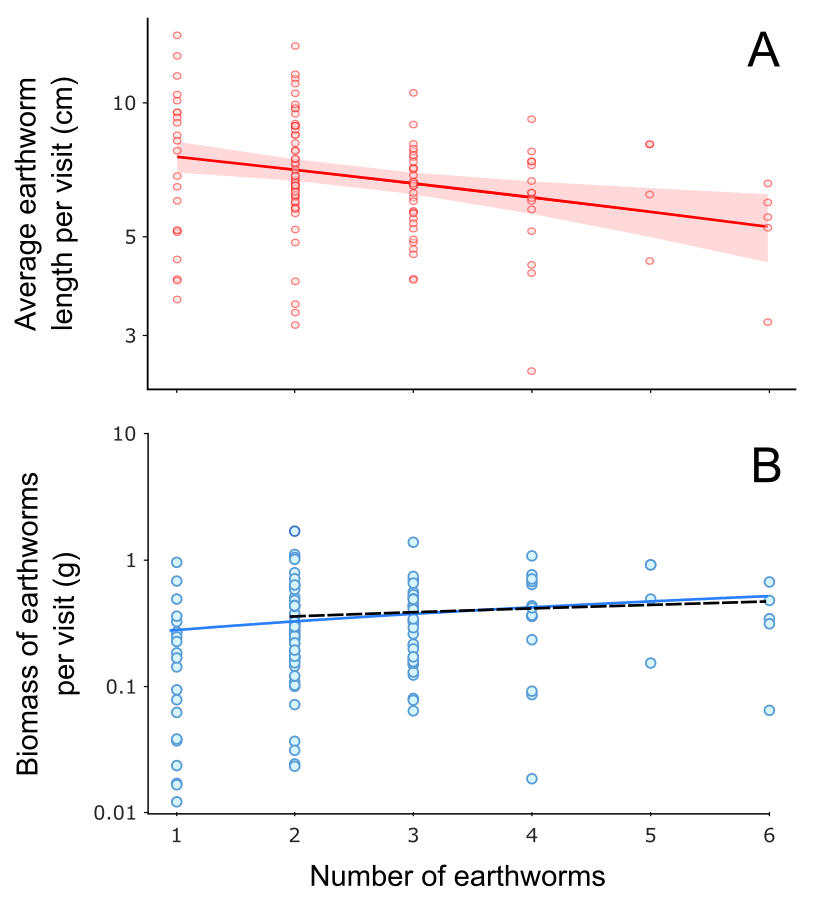

Supplement: Supplemental Information 3 — (A) Effect of the number of earthworms per visit on average earthworm length, with predicted values (red line) and confidence intervals (shaded area) from the lowest AICc model explaining variation in the response variable (Analysis 2 in Table 1; Table S5). In the LMER model, the average earthworm length per visit (y- axis) was square-root transformed to improve the normality of model residuals. (B) Positive correlation (Pearson r = 0.203, n = 128 visits, p ¡ 0.05; blue solid line: y = 0.049x + 0.227, R2 = 0.041) exists between the number of earthworms per visit (x) and the biomass of earthworms per visit (y). This relationship illustrates the positive correlation between the two variables representing distinct aspects (number vs. biomass) of earthworm amount during a single visit. However, this correlation is caused by lower y-values associated with visits featuring only one earthworm (x = 1), and it loses significance for x values between 2 and 6 (Pearson r = 0.104, n = 106 visits, p = 0.287; black dashed line: y = 0.029x + 0.299, R2 = 0.011). Both y-axes are log-scaled for visualization. [file peerj-12-17189-s003.png]

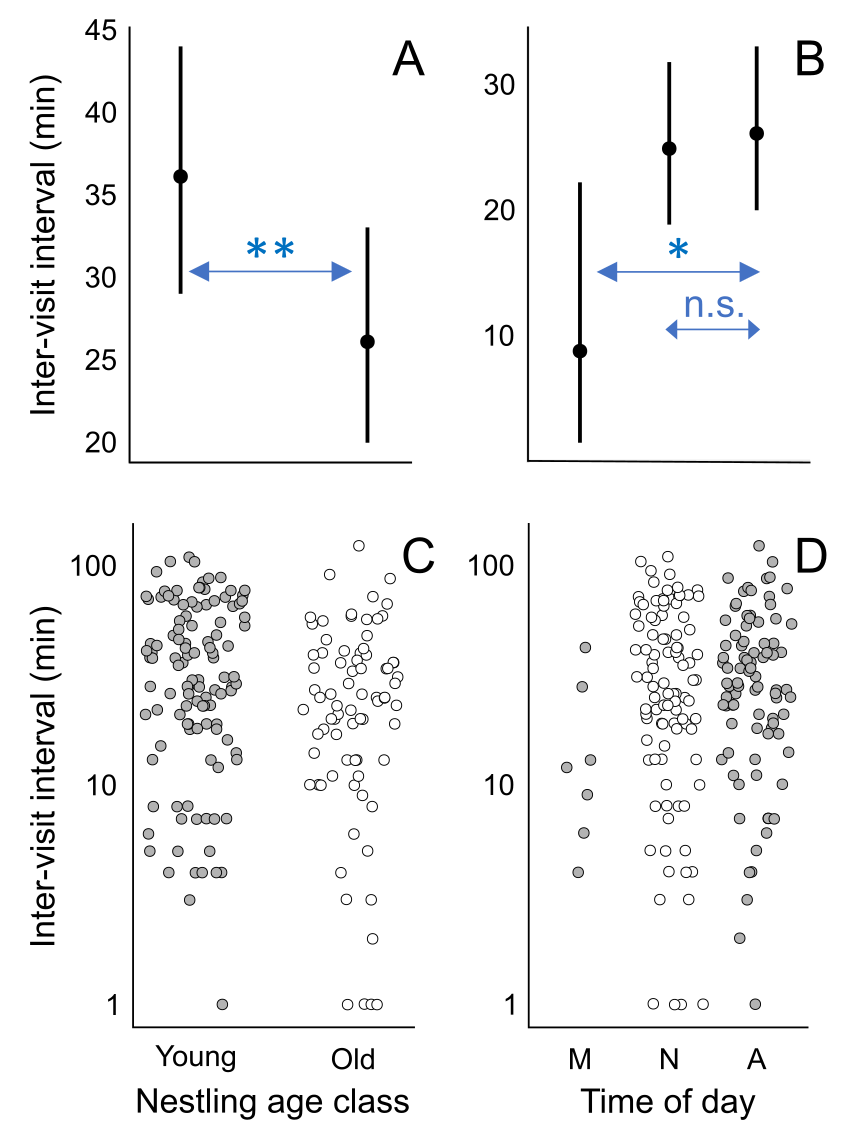

Supplement: Supplemental Information 4 — (A) Effect of nestling age class on the inter-visit interval. (B) Effect of time of day on the inter-visit interval. M, N, and A represent morning (8:00 to 10:00 h), noon (10:00 to 14:00 h), and afternoon (14:00 to 18:00 h), respectively. Panels A and B are derived from the model with the lowest AICc value that explains the variation in the response variable (Analysis 4 in Table 1; Table S10). In the statistical analysis, the response variable was square-root transformed to improve the normality of model residuals. Details of statistical analyses are shown in Table S10. The filled circles indicate predicted values of the inter-visit internal (in minutes), and vertical bars represent 95% confidence intervals. n.s. indicates non-significant; * indicates p ¡ 0.05; ** indicates p ¡ 0.01. (C) Raw data points of inter-visit intervals for young and old nestlings submitted to Analysis 4 (Table 1). (D) Raw data points of inter-visit intervals for morning, noon, and afternoon submitted to Analysis 4 (Table 1). In C and D, raw data points are jittered, and the y-axis is log-scaled for visualization. [file peerj-12-17189-s004.png]

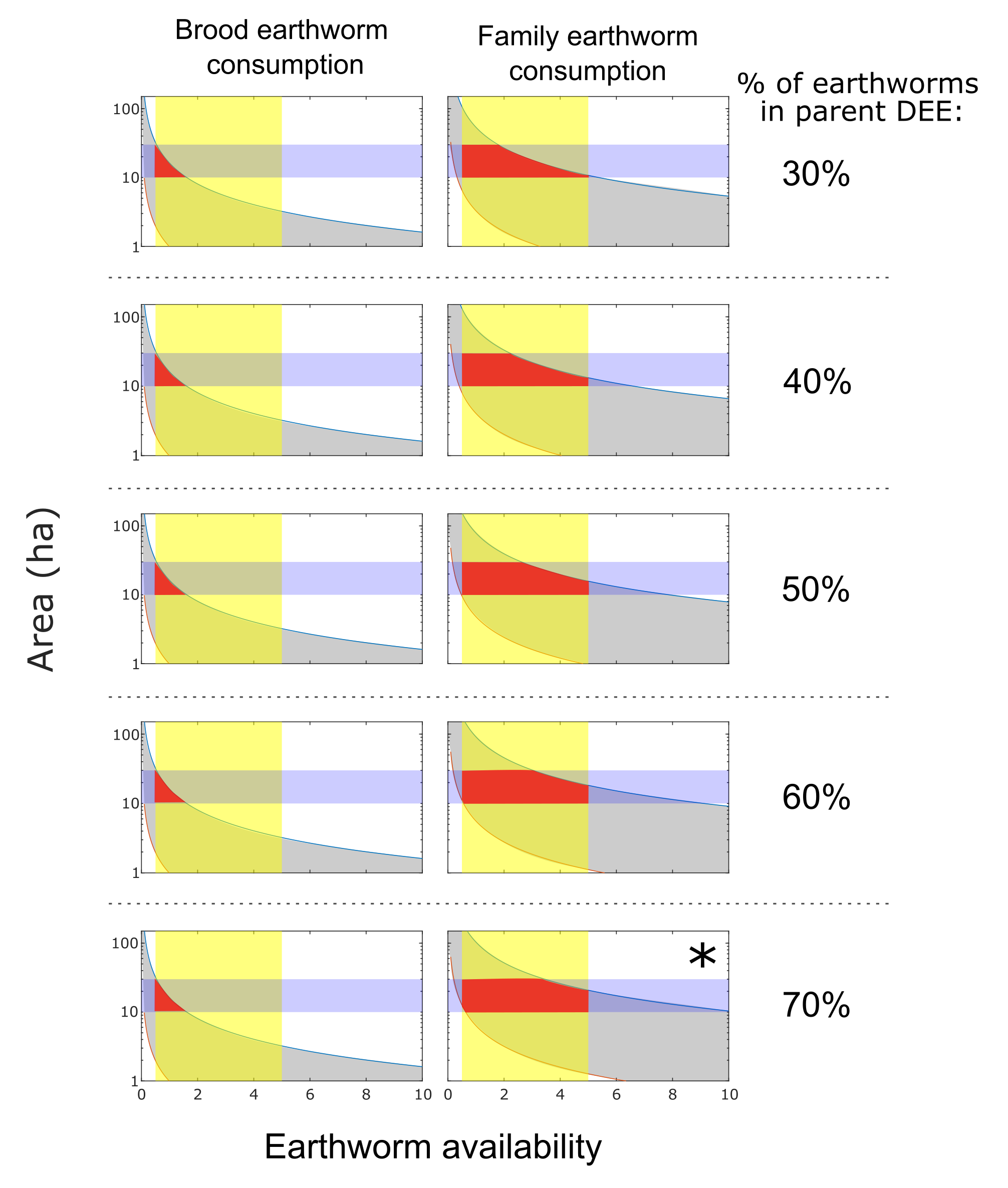

Supplement: Supplemental Information 5 — The gray band represents the estimated range of habitat area (vertical axis; ha, logarithmic scaled) that contains a number of earthworms to meet brood or family earthworm consumption. This estimation depends on the proportion (horizontal axis; percentage range 0–10% shown) of local earthworm density available to foraging pittas. The lower (orange) and upper (blue) edges of the gray band correspond to estimates calculated for the highest and lowest values, respectively, of epigeic earthworm densities in the fairy pitta habitats (Minamiya, Ishizuka & Tsukamoto, 2007; Kim et al., 2014). The vertical yellow band indicates the range (0.5–5%) on the horizontal axis likely to occur in nature based on Duriez, Ferrand & Binet (2006; see Methods ‘Assessing predicted and observed home range overlap’ section). The horizontal purple band indicates the estimated breeding home range size of the fairy pitta (10–30 ha). In each panel, the area marked with the red-shaded polygon indicates the ‘overlap’ region presenting predicted home range size that meets the observed home range size in the given earthworm availability (0.5–5%). The panels are arranged in two columns: brood or family earthworm consumption. The five rows represent different proportions of earthworms in the parent DEE. The panel marked with an asterisk is presented in Fig. 4A. The y-axis uses a logarithmic scale for visualization. [file peerj-12-17189-s005.png]

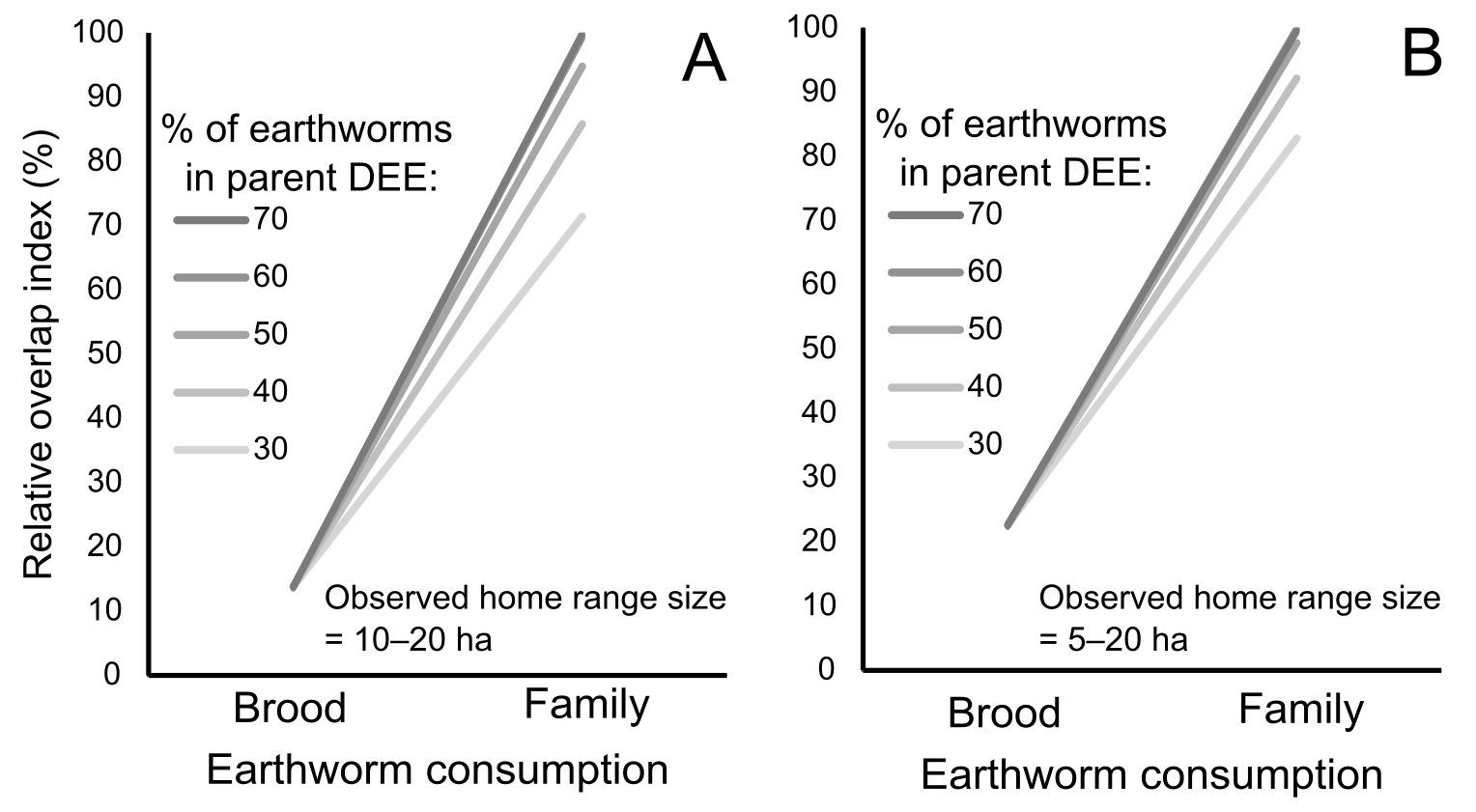

Supplement: Supplemental Information 6 — The figures show the relative overlap index for brood and family earthworm consumption. The calculations were based on an observed home range size of either 10–20 ha (A) or 5–20 ha (B). Y-axis indicates relative overlap index (%) with 100% indicating a theoretical maximum overlap. The five lines represent situations that differ in the % of earthworms in the parent DEE ranging from 30% to 70%. [file peerj-12-17189-s006.png]

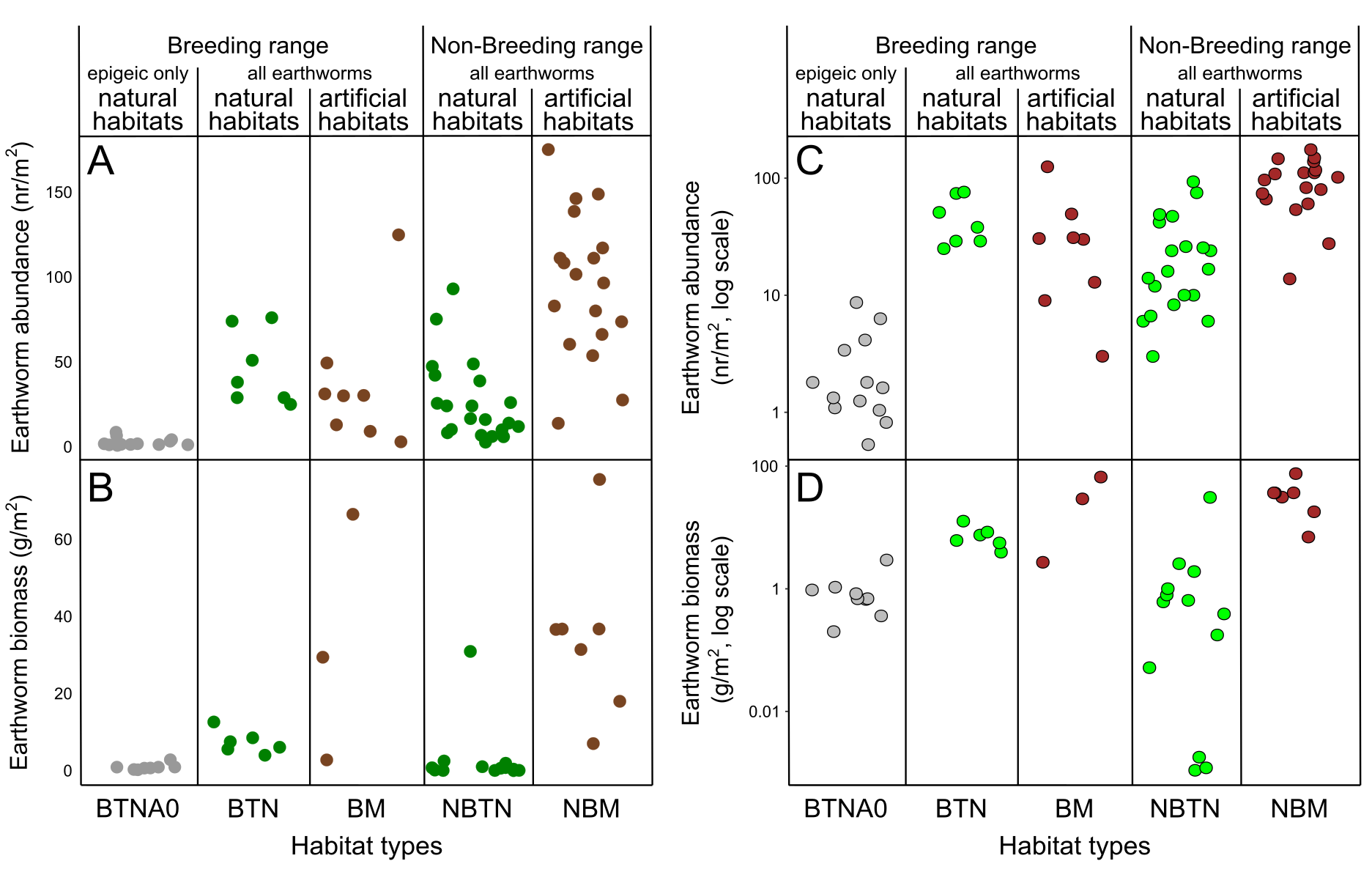

Supplement: Supplemental Information 7 — Habitat types: BTNA0 –Breeding range, in Typical Natural habitats, soil layer A0 only; BTN –Breeding range, in Typical Natural habitats; BM –Breeding range, in Modified habitats that may provide sufficient vegetation cover for pittas to safely forage there; NBTN –Non-Breeding range, in Typical Natural habitats; NBM –Non-Breeding range, in Modified habitats that may provide sufficient vegetation cover for pittas to safely forage there. Except for BTNA0, sampling was conducted from the soil surface to a variable depth deeper than the A0 layer. Data are tabularized in Data S1. (C, D) These are additional figures for A and B, using a logarithmic scale on the y-axes for visualization. [file peerj-12-17189-s007.png]
