# Supplementary material for: Assessing dietary specialization to inform the conservation of the fairy pitta (Pitta nympha), an endangered vermivore
Source: PeerJ. 2024 Apr 29;12:e17189. doi: 10.7717/peerj.17189 (PMC11064856; doi:10.7717/peerj.17189)
Supplement: Supplemental Information 8 [file peerj-12-17189-s008.pdf]

Table S1:
Information on hatching and fledging dates, the number of eggs, nestlings, and fledglings, as well as the collected video evidence for the four observed nests.
Fledging age is calculated by considering the hatching date as day 1.

Nest ID	Total duration of video recordings (hours)	Nest location	Hatching date	Fledging date	Fledging age	No. of eggs	No. of nestlings	No. of fledglings	No. of days with video [ages in days at which video was filmed] (number of hours/number of visits)	
									Young age (1–7 days old)	Old age (8–12 days old)
Nest 1	26.1	on rock	2012.06.30	.	NA	6	6	6	3 [3, 4, 5] (10.8/24)	3 [8, 9, 11] (15.3/27)
Nest 2	19.9	on slope	2012.07.06	2012.07.17	12	6	5	5	2 [4, 7] (8.6/8)	3 [9, 11, 12] (11.3/10)
Nest 3	76.9	on rock	2013.06.24	2013.07.06	13	5	5	5	7 [1–7] (50.5/78)	5 [8–12] (26.4/45)
Nest 4	25.3	on slope	2017.06.24	2017.07.06	13	6	6	6	6 [2–7] (14.8/24)	3 [8, 9, 10] (10.5/17)

Table S2:
Nestling diet composition.
Some arthropods could not be identified due to of the angle and lighting conditions in the recorded video. The data is summarized in Fig. 1C.

Prey type	Number of prey items					% of total prey number				
	Nest 1	Nest 2	Nest 3	Nest 4	All four nests	Nest 1	Nest 2	Nest 3	Nest 4	All four nests
Earthworms	117	40	252	138	547	88.0	81.6	81.5	88.5	84.5
Centipedes (Chilopoda)	3	6	18	7	34	2.3	12.2	5.8	4.5	5.3
Unidentified arthropods	5	1	3	1	10	3.8	2.0	1.0	0.6	1.5
Beetles (Coleoptera)	2	1	3	3	9	1.5	2.0	1.0	1.9	1.4
Planarians: *Bipalium* (Tricladia)			7	1	8	0.0	0.0	2.3	0.6	1.2
Caterpillars (Lepidoptera larvae)	2		2	3	7	1.5	0.0	0.6	1.9	1.1
Grasshoppers (Orthoptera)	1		6		7	0.8	0.0	1.9	0.0	1.1
Insect pupae	1		2	1	4	0.8	0.0	0.6	0.6	0.6
Mole crickets (Orthoptera)	1		3		4	0.8	0.0	1.0	0.0	0.6
Dragonfly (Odonata)		1			1	0.0	2.0	0.0	0.0	0.2
Moth (Lepidoptera adults)				1	1	0.0	0.0	0.0	0.6	0.2
Snake (Serpentes, Reptilia)				1	1	0.0	0.0	0.0	0.6	0.2
Stick insect (Phasmatodea)	1				1	0.8	0.0	0.0	0.0	0.2
Unidentified preys (not earthworm)			13		13	0.0	0.0	4.3	0.0	2.0
Total	133	49	309	156	647	100.0	100.0	100.0	100.0	100.0
Number of prey items per visit: range (mean ± SD):	1 – 5 (2.47 ± 1.06)	1 – 6 (2.44 ± 1.25)	1 – 6 (2.57 ± 1.14)	1 – 7 (3.73 ± 1.63)	1 – 7 (2.74 ± 1.31)					

Table S3:
Estimated number and biomass of earthworms consumed by nestlings of the fairy pitta from hatching to fledging (12.05 days), based on our empirical data.
See Methods 'Estimation of brood earthworm consumption' section for details of calculations. * – considering that the smallest amount of results for nest 2, we average the data from the three remaining nests (nest 1, 3, 4; each filmed for at least 25 hours) to obtain a more accurate estimate of brood earthworm consumption. & – based on this number, we subsequently estimate the number of earthworms consumed by a pair of adults and their brood (Table S12).

Nest ID, the number of hours and days of video recording	Number of earthworms consumed by a nestling per day	Number of earthworms consumed by a nestling from hatching to fledging	Biomass of earthworms consumed by a nestling from hatching to fledging	Estimated number and biomass of earthworms consumed by a brood		
				Number	Ash-free dry mass (g)	Estimated fresh mass (g)
Nest 1, 26.1 video-hours during 6 days, including 3 days for nestlings < 7 days old	81	163	29.0	976	174.3	1009.2
Nest 2 19.9 video-hours during 5 days, including 1 day for nestlings < 7 days old	41	83	12.2	495	73.1	423.5
Nest 3 76.9 video-hours during 12 days, including 6 days for nestlings < 7 days old	51	124	13.7	619	68.7	397.6
Nest 4 25.3 video-hours during 9 days, including 5 days for nestlings < 7 days old	113	227	19.9	1359	119.2	690.2
Average from nests 1, 2, 3, 4	71.6	148.9	18.7	862.4	108.8	630.1
**Average from nests 1, 3, 4** *	81.7	**171.0** &	20.9	984.9	120.7	699.0

Table S4:

The top (within ΔAICc of 2) models explaining variation in the number of prey items (or number of earthworms).

The initial models included earthworms present (or only earthworms), inter-visit interval, nestling age class, and time of day as fixed effects. Nest ID was used as a random effect in the main set of analyses (GLMER). As the dependent variable is a count, we applied Conway–Maxwell–Poisson distribution with log link function. The values show the effect estimate for the variables, standard error in parentheses (± SE), (Z-value), and *p*-value. Blank cells indicate that the variable was not included in the model. Significant effects are shown in bold, except for intercept. The models in the alternative analysis with nest ID as fixed effect (GLM) resulted in similar effect estimates and conclusions. The table concerns the results presented in Figs. 2B and S2. The hypotheses tested in these analyses are listed in Table 1: Analysis 1. Sample sizes: 200 visits in 4 nests (Number of prey items analysis using "earthworms present" variable), 192 visits in 4 nests (Number of prey items analysis using "only earthworms" variable), and 192 visits in 4 nests (Number of earthworms analysis using "only earthworms" variable).

Response variable	Feeding visit type variable used in model	Model type	Model ID	Intercept	Earthworms present; estimate for "NoE"	Only earthworms; estimate for "MIX"	Inter-visit interval	Nestling age class; estimate for "young"	Time of day	Nest ID; estimate for "2 (left)",	"3 (middle)",	"4 (right)"	*df*	logLik	AICc	ΔAICc
No. of prey items	earth-worms present n=200 visits	GLMER	m1	1.060 ± 0.084, (12.572), < 2E-16	**-1.031** ± 0.276, (-3.738), 0.0002								4	-310.442	629.1	0
			m2	1.040 ± 0.094, (11.108), < 2E-16	**-1.030** ± 0.276, (-3.736), 0.0002		0.001 ± 0.001, (0.480), 0.631						5	-310.327	631.0	1.87
		GLM	m1	0.956 ± 0.065, (14.710), < 2E-16	**-1.023** ± 0.273, (-3.734), 0.0002					-0.019 ± 0.143, (-0.132), 0.895	0.031 ± 0.076, (0.410), 0.682	**0.380** ± 0.089, (4.291), 1.78E-05	6	-304.791	622.0	0
			m2	0.940 ± 0.073, (12.838), < 2E-16	**-1.022** ± 0.274, (-3.731), 0.0002		0.001 ± 0.001 (0.464), 0.643			-0.026 ± 0.144, (-0.183), 0.855	0.028 ± 0.077, (0.367), 0.713	**0.376** ± 0.089, (4.221), 2.43E-05	7	-304.684	624.0	1.93
	only earth-worms n=192 visits	GLMER	m1	0.975 ± 0.079, (12.33), < 2E-16		**0.276** ± 0.059, (4.65), 3.31E-06							4	-294.826	597.9	0
			m2	0.947 ± 0.088, (10.815), < 2E-16		**0.286** ± 0.060, (4.712), 2,46E-06		0.046 ± 0.060, (0.769), 0.442					5	-294.529	599.4	1.52
		GLM	m1	0.882 ± 0.066, (13.364), < 2E-16		**0.270** ± 0.059, (4.570), 4.88E-06				0.052 ± 0.142, (0.369), 0.712	-0.008 ± 0.075, (-0.103), 0.917	**0.338** ± 0.087, (3.876), 0.0001	6	-289.555	591.6	0
			m2	0.856 ± 0.073, (11.640), < 2E-16		**0.281** ± 0.061, (4.634), 3.59E-06		0.047 ± 0.060, (0.789), 0.430		0.057 ± 0.142, (0.403), 0.789	-0.017 ± 0.076, (-0.235), 0.814	**0.335** ± 0.087, (3.834), 0.0001	7	-289.243	593.1	1.53
No. of worms	only earth-worms n=192 visits	GLMER	m1	0.983 ± 0.094, (10.412), < 2E-16		**-0.174** ± 0.072, (-2.424), 0.015							4	-287.574	583.4	0
			m2	0.936 ± 0.104, (8.995), < 2E-16		**-0.157** ± 0.073, (-2.156), 0.031		0.077 ± 0.068, (1.134), 0.257					5	-286.928	584.2	0.82
			m3	0.964 ± 0.104, (9.256), < 2E-16		**-0.175** ± 0.072, (-2.445), 0.015		0.001 ± 0.001, (0.441), 0.659					5	-287.477	585.3	1.91
		GLM	m1	0.877 ± 0.071, (12.276), < 2E-16		**-0.178** ± 0.071, (-2.508), 0.012				0.057 ± 0.148, (0.386), 0.700	-0.029 ± 0.083, (-0.351), 0.726	**0.407** ± 0.096, (4.233), 2.3E-05	6	-281.887	576.2	0
			m2	0.831 ± 0.082, (10.162), < 2E-16		**-1.161** ± 0.073, (-2.221), 0.026		0.078 ± 0.067, (1.160), 0.246		0.068 ± 0.147, (0.461), 0.650	-0.041 ± 0.084, (-0.492), 0.623	**0.403** ± 0.096, (4.213), 2.53E-05	7	-281.211	577.0	0.80
			m3	0.863 ± 0.080, (10.771), < 2E-16		**-0.180** ± 0.071, (-2.528), 0.012	0.001 ± 0.001, (0.407), 0.684			0.050 ± 0.149, (0.336), 0.737	-0.033 ± 0.084, (-0.387), 0.699	**0.402** ± 0.097, (4.168), 3.07E-05	7	-281.805	578.2	1.99

Table S5:

The top (within ΔAICc of 2) models explaining variation in average earthworm length per visit for visits with only earthworms (OnlyE).

The initial model included the number of earthworms, nestling age class, time of day, and inter-visit interval as fixed effects. Nest ID was used as a random effect in the LMER analyses. The response variable was square-root transformed to improve the normality of model residuals. The values show effect estimates for the variables, standard error in parentheses (± SE), (t-value), and *p*-value. Blank cells indicate that the variable was not included in the model. Significant effects are shown in bold, except for the intercept. The models in the additional analysis with nest ID as a fixed effect (LM analyses) resulted in very similar effect estimates and conclusions. The table concerns the results presented in Fig. S3A. The hypotheses evaluated in this analysis are listed in Table 1: Analysis 2. Sample size: 128 "OnlyE" visits in 4 nests.

Model type	Model ID	Intercept	Number of earthworms	Nestling age class; estimate for "young"	Time of day; estimate for "morning" and noon"	Inter-visit interval	Nest ID; estimate for "2 (left)", "3 (middle)", "4 (right)"			*df*	logLik	AICc	ΔAICc
LMER	m1	2.841 ± 0.080, (35.480), < 2E-16	**-0.091** ± 0.028, (-3.224), 0.001							4	-64.734	137.8	0
LM	m1	2.768 ± 0.099, (27.819), < 2E-16	**-0.092** ± 0.029, (-3.186), 0.001				0.029 ± 0.136, (0.216), 0.829	0.112 ± 0.084, (1.336), 0.184	0.108 ± 0.115, (0.944), 0.347	3	-59.625	125.4	0
	m2	2.735 ± 0.107, (25.433), < 2E-16	**-0.096** ± 0.029, (-3.268), 0.001	0.061 ± 0.074, (0.823), 0.412			0.043 ± 0.137, (0.319), 0.750	0.112 ± 0.084, (1.336), 0.183	0.114 ± 0.115, (0.993), 0.322	4	-59.260	126.8	1.40

Table S6:

The top (within ΔAICc of 2) models explaining variation in feeding visit types in two analyses: one for “Earthworms present” response variable and another for “Only earthworms” response variable. Nestling age is represented categorically as either young or old in both analyses.

The initial models included nestling age class, inter-visit interval, and time of day as fixed effects. Due to rarity of NoE visits (earthworms present) in the morning (time of day), the time of day was only included in the initial model explaining the variation in only earthworms. Nest ID was used as a random effect in the main set of analyses (GLMER). As the dependent variable is binary (either YesE = 0 and NoE = 1, or OnlyE = 0 and MIX = 1), we applied Binomial distribution with logit link function, and the models estimate the effect of fixed effects on the probability of NoE (earthworms present) or MIX (only earthworms). The values show effect estimates for the variables, standard error in parentheses (± SE), (Z-value), and *p*-value. Blank cells indicate that the variable was not included in the model. Significant effects are shown in bold, except for intercept. The models in the additional analysis with nest ID as fixed effect (GLM) resulted in similar effect estimates and conclusions. The table concerns the results presented in Fig. 2D, E. The hypotheses tested in these analyses are listed in Table 1: Analysis 3. Sample size: 200 visits in 4 nests (Earthworms present analysis) and 192 visits in 4 nests (Only earthworms analysis).

Dependent variable	Model type	Model ID	Intercept	Nestling age class; estimate for “young”	Inter-visit interval (min)	Time of day; estimate for “morning (left)” and noon (right)”	Nest ID; estimate for “2 (left)”, “3 (middle)”, “4 (right)”			*df*	logLik	AICc	ΔAICc
Earthworms present [probability of NoE]	GLMER	m1	-2.410 ± 0.394, (-6.110), 9.96E-10	**-2.325** ± 1.079, (-2.155), 0.031						3	-29.921	66.0	0
	GLM	m1	-2.410 ± 0.394, (-6.110), 9.96E-10	**-2.325** ± 1.079, (-2.155), 0.031						2	-29.921	63.9	0
		m2	-2.520 ± 0.605, (-4.172), 3.02E-05	**-2.359** ± 1.088, (-2.167), 0.030	0.003 ± 0.014, (0.247), 0.804					3	-29.892	65.9	2.00
Only earthworms [probability of MIX]	GLMER	m1	-0.510 ± 0.458, (-1.112), 0.266	**-0.889** ± 0.327, (-2.719), 0.006						3	-118.015	242.2	0
		m2	-0.765 ± 0.512, (-1.494), 0.135	**-0.983** ± 0.338, (-2.904), 0.003	0.008 ± 0.006, (1.293), 0.196					4	-117.181	242.6	0.42
	GLM	m1	-0.728 ± 0.392, (-1.856), 0.063	**-0.941** ± 0.392, (-1.856), 0.004			-16.444 ± 1152.282, (-0.014), 0.988	**0.848** ± 0.427, (1.982), 0.047	0.828 ± 0.523, (1.583), 0.113	5	-111.687	233.7	0
		m2	-0.951 ± 0.429, (-2.216), 0.026	**-1.039** ± 0.340, (-3.057), 0.002	0.008 ± 0.006, (1.352), 0.176		-16.540 ± 1153.000 (-0.014), 0.988	0.824 ± 0.432, (1.907), 0.056	0.779 ± 0.526, (1.480), 0.138	6	-110.773	234.0	0.30

Table S7:

The top (within ΔAICc of 2) models explaining variation in feeding visit types in two analyses: one for "Earthworms present" response variable and another for "Only earthworms" response variable. Nestling age is represented in days (range: 1–12).

In the analyses presented in Table S7, one of the fixed effects was the nestling age class, but here, nestling age (days) was used instead of nestling age class. The initial models included nestling age class, inter-visit interval, and time of day as fixed effects. Due to rarity of NoE visits (earthworms present: NoE) in the morning (time of day), the time of day was only included in the initial model explaining the variation in only earthworms (OnlyE vs MIX). Nest ID was used as a random effect in the main analyses (GLMER). As the dependent variable is binary (either YesE = 0 and NoE = 1 for "earthworms present" variable, or OnlyE = 0 and MIX = 1 for "only earthworms" variable), we applied Binomial distribution with logit link function, and the models estimate the effect of fixed effects on the probability of NoE (earthworms present) or MIX (only earthworms). The values show effect estimates for the variables, standard error in parentheses (± SE), (Z-value), and *p*-value. Blank cells indicate that the variable was not included in the model. Significant effects are shown in bold, except for intercept. The models in the additional analysis with nest ID as fixed effect (GLM) resulted in similar effect estimates and conclusions. The table concerns the results presented in Fig. S1. The hypotheses tested in these analyses are listed in Table 1: Analysis 3. Sample size: 200 visits in 4 nests (Earthworms present analysis) and 192 visits in 4 nests (Only earthworms analysis).

Dependent variable	Model type	Model ID	Intercept	Nestling age (days)	Inter-visit interval (min)	Time of day; estimate for "morning (left)" and noon (right)"	Nest ID; estimate for "2 (left)", "3 (middle)", "4 (right)"			*df*	logLik	AICc	ΔAICc
Earthworms present [probability of NoE]	GLMER	m1	-8.071 ± 2.165, (-3.727), 0.0001	**0.554** ± 0.207, (2.672), 0.007						3	-27.016	60.2	0
	GLM	m1	-8.071 ± 2.165, (-3.727), 0.0001	**0.554** ± 0.207, (2.672), 0.007						2	-27.016	58.1	0
Only earthworms [probability of MIX]	GLMER	m1	-1.805 ± 0.600, (-3.006), 0.002	**0.126** ± 0.053, (2.349), 0.018						3	-118.663	243.5	0
		m2	-2.176 ± 0.697, (-3.121), 0.001	**0.139** ± 0.055, (2.517), 0.011	0.007 ± 0.006, (1.200), 0.230					4	-117.946	244.1	0.65
	GLM	m1	-2.176 ± 0.555, (-3.920), 8.86E-05	**0.139** ± 0.054, (2.574), 0.010			-16.513 ± 1211.757, (-0.014), 0.989	0.791 ± 0.424, (1.863), 0.062	0.878 ± 0.522, (1.683), 0.092	5	-112.445	235.2	0
		m2	-2.532 ± 0.634, (-3.992), 6.54E-05	**0.153** ± 0.055, (2.754), 0.005	0.008 ± 0.006, (1.278), 0.201		-16.610 ± 1209.000, (-0.014), 0.989	0.763 ± 0.428, (1.781), 0.074	0.833 ± 0.524, (1.587), 0.112	6	-111.629	235.7	0.50

Table S8:

The effect of nestling age class on earthworm frequency in nestling diet at each nest.

The *p*-values were calculated using a one-tailed Fisher's exact test to test the hypothesis: "Young > Old" within each nest. Fisher's combined probability test was then performed using these one-tailed *p*-values.

Nest ID	Prey type	Nestling age class		Fisher's exact test *p*-value
		Young (1 to 7 days)	Old (8 to 12 days)	
Nest 1	Earthworms	75	42	< 0.0001
	Others	0	16	
	% of earthworms	100.0	72.4	
Nest 2	Earthworms	24	16	0.313
	Others	4	5	
	% of earthworms	85.7	76.2	
Nest 3	Earthworms	158	94	0.155
	Others	31	26	
	% of earthworms	83.6	78.3	
Nest 4	Earthworms	81	57	0.324
	Others	9	9	
	% of earthworms	90.0	86.4	
Total	Earthworms	350	210	Fisher combined chi-square = 37.533,
	Others	32	55	*df* = 8, *p* < 0.0001
	% of earthworms	91.6	79.2	

Table S9:

The effect of nestling age class on feeding visit type: differences between nestling age classes in the frequency of visits with only earthworms per nest.

The *p*-values were calculated using a one-tailed Fisher's exact test to test the hypothesis: "Young > Old" within each nest. Fisher's combined probability test was then performed using these one-tailed *p*-values.

Nest ID	Feeding visit type	Nestling age class: Young (1 to 7 days)	Nestling age class: Old (8 to 12 days)	Fisher's exact test *p*-value
Nest 1	Only earthworms (OnlyE)	24	13	< 0.0001
	No earthworms or mixed (NoE, MIX)	0	14	
	**% visits with earthworms only (OnlyE/[OnlyE+NoE+MIX])**	100.0	48.1	
Nest 2	Only earthworms (OnlyE)	6	8	0.794
	No earthworms or mixed (NoE, MIX)	2	2	
	**% visits with earthworms only (OnlyE/[OnlyE+NoE+MIX])**	75.0	80.0	
Nest 3	Only earthworms (OnlyE)	27	20	0.898
	No earthworms or mixed (NoE, MIX)	51	25	
	**% visits with earthworms only (OnlyE/[OnlyE+NoE+MIX])**	34.6	44.4	
Nest 4	Only earthworms (OnlyE)	16	9	0.286
	No earthworms or mixed (NoE, MIX)	8	8	
	**% visits with earthworms only (OnlyE/[OnlyE+NoE+MIX])**	66.7	52.9	
Total	Only earthworms (OnlyE)	73	50	Fisher combined chi-square = 25.324, *df* = 8, *p* = 0.001
	No earthworms or mixed (NoE, MIX)	61	49	
	**% visits with earthworms only (OnlyE/[OnlyE+NoE+MIX])**	54.5	50.1	

Table S10:
The top (within ΔAICc of 2) models explaining variation in the inter-visit interval.

The initial model included nestling age class, time of day, only earthworms (OnlyE or MIX), rainfall category, and the number of prey items as fixed effects. Nest ID was used as a random effect in the main set of analyses (LMER). The response variable was square-root transformed to improve the normality of model residuals. The values show the effect estimate for the variables, standard error in parentheses (± SE), (t-value), and *p*-value. Blank cells indicate that the variable was not included in the model. Significant effects are shown in bold, except for intercept. The model in the additional analysis with nest ID as fixed effect (LM) resulted in similar effect estimates and conclusions. The table concerns the results presented in Fig. S4. The hypotheses tested in this analysis are listed in Table 1: Analysis 4. Sample size: 192 intervals (preceding YesE visits) in 4 nests.

Model type	Model ID	Intercept	Nestling age class; estimate for "young"	Time of day; estimate for "morning (left)" and noon (right)"		Only earthworms; estimate for "MIX"	Rainfall category; estimate for "light rain"	Number of prey items	Nest ID; estimate for "2 (left)", "3 (middle)", "4 (right)"			*df*	logLik	AICc	ΔAICc
LMER	m1	5.107 ± 0.326, (15.663), 8.35E-07	**0.901** ± 0.325, (2.766), 0.006	**-2.149** ± 0.871, (-2.466), 0.014	-0.119 ± 0.324, (-0.369), 0.712							6	-442.204	856.9	0
	m2	4.849 ± 0.377, (12.850), 1.07E-08	**0.683** ± 0.363, (1.881), 0.061	**-2.255** ± 0.873, (-2.582), 0.010	-0.120 ± 0.323, (-0.372), 0.710		0.532 ± 0.395, (1.346), 0.179					7	-421.308	857.2	0.36
	m3	4.928 ± 0.352, (13.964), 1.44E-07	**0.977** ± 0.331, (2.946), 0.003	**-2.192** ± 0.871, (-2.516), 0.012	-0.111 ± 0.324, (-0.343), 0.731	0.408 ± 0.343, (1.188), 0.236						7	-421.649	857.9	1.04
	m4	4.634 ± 0.403, (11.490), 1.18E-09	**0.751** ± 0.366, (2.052), 0.041	**-2.306** ± 0.872, (-2.643), 0.008	-0.110 ± 0.323, (-0.342), 0.732	0.445 ± 0.343, (1.297), 0.196	0.570 ± 0.396, (1.44), 0.151					8	-420.622	858.0	1.17
	m5	4.977 ± 0.377, (13.175), 5.78E-09		**-2.166** ± 0.877, (-2.468), 0.014	-0.076 ± 0.325, (-0.236), 0.813		**0.864** ± 0.356, (2.425), 0.016					6	-422.973	858.4	1.54
LM	m1	5.081 ± 0.294, (17.267), < 2E-16	**0.908** ± 0.326, (2.788), 0.005	**-2.104** ± 0.871, (-2.418), 0.016	-0.106 ± 0.325, (-0.327), 0.744							5	-421.703	853.7	0
	m2	4.832 ± 0.347, (13.892), < 2E-16	0.696 ± 0.362, (1.920), 0.056	**-2.200** ± 0.871, (-2.525), 0.012	-0.107 ± 0.324, (-0.330), 0.741		0.526 ± 0.395, (1.920), 0.184					6	-420.798	854.1	0.32
	m3	4.895 ± 0.330, (14.820), < 2E-16	**0.983** ± 0.331, (2.970), 0.003	**-2.163** ± 0.870, (-2.486), 0.013	-0.100 ± 0.324, (-0.309), 0.757	0.420 ± 0.342, (1.228), 0.221						6	-420.932	854.3	0.59
	m4	4.609 ± 0.384, (11.978), < 2E-16	**0.760** ± 0.364, (2.083), 0.038	**-2.272** ± 0.871, (-2.609), 0.009	-0.100 ± 0.323, (-0.311), 0.756	0.460 ± 0.460, (1.344), 0.180	0.569 ± 0.396, (1.438), 0.151					7	-419.870	854.3	0.62
	m5	4.712 ± 0.459, (10.266), < 2E-16	**0.918** ± 0.326, (2.817), 0.005	**-2.090** ± 0.869, (-2.411), 0.016	-0.120 ± 0.325, (-0.371), 0.711			0.130 ± 0.124, (1.048), 0.296				6	-421.142	854.7	1.01
	m6	4.536 ± 0.403, (11.242), < 2E-16	**0.910** ± 0.327, (2.783), 0.005	**-2.180** ± 0.875, (-2.783), 0.013	-0.139 ± 0.325, (-0.428), 0.669				1.428 ± 0.741, (1.927), 0.055	0.619 ± 0.404, (1.533), 0.127	0.818 ± 0.516, (1.584), 0.115	8	-419.145	855.1	1.35
	m7	5.003 ± 0.251, (19.895), < 2E-16	**0.818** ± 0.326, (2.508), 0.013									3	-424.660	855.4	1.72
	m8	4.583 ± 0.472, (9.698), < 2E-16	**0.730** ± 0.472, (9.698), 0.047	**-2.182** ± 0.872, (-2.501), 0.013	-0.117 ± 0.324, (-0.363), 0.717		0.458 ± 0.405, (1.131), 0.259	0.099 ± 0.127, (0.782), 0.435				7	-420.483	855.6	1.85
	m9	4.377 ± 0.421, (10.381), < 2E-16	**0.998** ± 0.333, (2.992), 0.003	**-2.214** ± 0.874, (-2.531), 0.012	-0.126 ± 0.325, (-0.389), 0.697	0.446 ± 0.350, (1.274), 0.204			1.541 ± 0.745, (2.068), 0.040	0.545 ± 0.407, (1.337), 0.182	0.739 ± 0.519, (1.425), 0.155	9	-418.302	855.6	1.86
	m10	4.977 ± 0.342, (14.553), < 2E-16		**-2.083** ± 0.875, (-2.380), 0.018	-0.061 ± 0.325, (-0.189), 0.850		**0.861** ± 0.357, (2.410), 0.016					5	-422.672	855.7	1.94
	m11	4.293 ± 0.448, (9.573), < 2E-16	**0.703** ± 0.367, (1.916), 0.056	**-2.289** ± 0.879, (-2.605), 0.009	-0.142 ± 0.325, (-0.437), 0.662		0.493 ± 0.400, (1.235), 0.218		1.318 ± 0.745, (1.768), 0.078	0.655 ± 0.404, (1.620), 0.106	0.833 ± 0.516, (1.615), 0.108	9	-418.353	855.7	1.97

Table S11:
The top (within ΔAICc of 2) models explaining variation in the number of earthworms, the biomass of a single earthworm, and the biomass of earthworms per visit in visits with only earthworms.
The initial models included rainfall category, inter-visit interval, and nestling age class as fixed effects. Nest ID was used as random effect, and for models involving the biomass of a single earthworm as a dependent variable, visit ID nested within nest ID was also used as random effect. As the number of earthworms is a count, we used GLMER and applied a Poisson distribution with the log-link function. LMER was used to explain the variation in the biomass of a single earthworm and the biomass of all earthworms per visit. The biomass of a single earthworm was box-cox transformed (exponential value of 0.1), and the biomass of all earthworms per visit is square-root transformed to improve the normality of model residuals. The values show the effect estimate for the variables, standard error in parentheses (± SE), (Z-value or t-value), and *p*-value. Blank cells indicate that the variable was not included in the model. Significant effects are shown in bold, except for intercept. The models in the additional analysis with nest ID as fixed effect (GLM or LM) resulted in similar effect estimates conclusions. The table concerns the results presented in Fig. 3. The hypotheses tested in these analyses are listed in Table 1: Analysis 5–7. Samples sizes are 328 earthworms and 128 visits (OnlyE visits) from 4 nests.

Dependent variable	Model type	Model ID	Intercept	Rainfall category; estimate for “light rain”	Inter-visit interval	Nestling age class; estimate for “young”	Nest ID; estimate for “2 (left)”, “3 (middle)”, “4 (right)”			*df*	logLik	AICc	ΔAICc
Number of earthworms	GLMER	m1	0.733 ± 0.103, (7.090), 1.34E-12	**0.293** ± 0.099, (2.948), 0.003						4	-195.314	399.0	0
		m2	0.691 ± 0.112, (6.162), 7.19E-10	**0.281** ± 0.100, (2.818), 0.005	0.001 ± 0.002, (0.919), 0.357					5	-194.897	400.3	1.33
	GLM	m1	0.655 ± 0.112, (5.839), 5.26E-09	**0.311** ± 0.098, (3.176), 0.001			-0.005 ± 0.151, (-0.033), 0.974	-0.014 ± 0.095, (-0.144), 0.886	0.304 ± 0.117, (2.604), 0.009	6	-191.418	395.5	0
		m2	0.627 ± 0.117, (5.359), 8.36E-08	**0.301** ± 0.098, (3.065), 0.002	0.001 ± 0.002, (0.839), 0.402		-0.023 ± 0.152, (-0.150), 0.881	-0.024 ± 0.095, (-0.254), 0.800	0.290 ± 0.118, (2.456) 0.014	7	-191.071	397.1	1.55
Biomass of a single earthworm	LMER (Nest ID /Visit ID)	m1	0.076 ± 0.006, (122.7), < 2E-16							4	298.070	-588.0	0
		m2	0.791 ± 0.013, (59.264), < 2E-16	**-0.043** ± 0.014, (-2.919), 0.004						5	298.934	-587.7	0.33
	LMER (Visit ID)	m1	0.756 ± 0.006, (122.7), < 2E-16							3	298.070	-590.1	0
		m2	0.791 ± 0.013, (59.254), < 2E-16	**-0.043** ± 0.014, (-2.919), 0.004						4	298.934	-589.7	0.32
Biomass of all earthworms per visit	LMER	m1	0.544 ± 0.021, (25.79), < 2E-16							3	-0.712	7.6	0
	LM	m1	0.500 ± 0.035, (13.976), < 2E-16			0.066 ± 0.044, (1.509), 0.134				3	3.377	-0.6	0
		m2	0.527 ± 0.044, (11.725), < 2E-16	-0.055 ± 0.056, (-0.983), 0.327		0.091 ± 0.050, (1.797), 0.07				4	3.869	0.6	1.15

Table S12:
Estimated number of earthworms consumed by a brood or family
These estimates are used to calculate the "Predicted home range", which provides the number of earthworms to meet brood (Methods 'Estimation of brood earthworm consumption' section) or family earthworm consumption (Methods 'Estimation of parent earthworm consumption for calculating family earthworm consumption' section). This calculation depends on the % of earthworms (% of the total abundance of epigeic earthworms) available to foraging birds on (Fig. S5).
* – This specific estimate of the number of earthworms is used to calculate the predicted home range size in Fig. 4A, and it is also marked with an asterisk (*) in Fig. S5.

% of earthworms in parents' daily energy expenditure	Earthworm consumption category: Brood or family	Number of earthworms consumed
30	Brood	855
	Family	2844
40	Brood	855
	Family	3508
50	Brood	855
	Family	4171
60	Brood	855
	Family	4834
70	Brood	855
	Family	**5497***
