# Supplementary material for: Assessing dietary specialization to inform the conservation of the fairy pitta (Pitta nympha), an endangered vermivore
Source: PeerJ. 2024 Apr 29;12:e17189. doi: 10.7717/peerj.17189 (PMC11064856; doi:10.7717/peerj.17189)
Supplement: Supplemental Information 10 [file peerj-12-17189-s010.pdf]

Supplementary Methods

Details of Methods part 9

1) Observations by Okada (1999 cited in Kim, 2010; Article S1) indicate that breeding birds are mainly found within 100–400 m from their nests. Assuming a circular home range, this estimated range would be 3–50 ha. Based on the assumption of a radius of approximately 250 m (midrange between 100 and 400), the hypothetical circular home range would comprise approximately 20 ha. Indeed, Okada (1999 cited in Kim, 2010; Article S1) suggested that there are approximately five pairs per square kilometer, which indicates a maximum home range size of ~20 ha (corresponding to r = 253 m of a hypothetical circular home range). However, if the birds do not use the entire area of the theoretical circle around their nest, then the foraging area may differ from that of a theoretical circle, depending on the direction and distance of their foraging trips from the nest.

2) We used approximate calculations based on transects to estimate the abundance of pittas in the relatively non-fragmented Chinese forests (Jiang et al., 2017). The transect method relies solely on the location and number of territorial vocalizations detected within a 200 m wide band of forest in response to playbacks of the Fairy Pitta's call. We chose two transects in Nonggang and three transects from the Mulun areas in Table 1 in Jiang et al. (2017), because only those transects contained fairy pittas. The transect lengths were 2.3 and 1.9 km and 4.6, 4.7, and 5.7 km for Nonggang and Mulun, respectively, and the number of individuals detected were 2 and 2 and 4, 6, and 2, respectively (Jiang et al., 2017). We calculated the estimated home range size of a pair of fairy pittas separately for each transect using the following equations (reasoning for using these equations is given below):

Home range size estimate 1 = area of the transect / (no. of individuals recorded / 2)
Home range size estimate 2 = area of the transect / (no. of individuals recorded)

Both estimates assume that all birds were paired. Home range size estimate 1 assumes that all individuals, both males and females, respond with vocalizations as long as they are present within the 200 m wide band of the transect. It also assumes that each vocalizing bird is distinguished from another by the observer. Home range size estimate 2, on the other hand, assumes that only males (and all males) respond to the playbacks used on transects, or generally that one member of a pair responded (or both responded but were at the same location and the observer could not determine whether the calls came from 2 different birds). We further calculated the average value of estimate 1 (22.6 ha) and that of estimate 2 (45.3 ha). If these estimates are correct, the breeding home range size of the Fairy Pitta varies from 22.6 to 45.3 ha, which corresponds to r_1 = 268 m and r_2 = 380 m, respectively, for the radius of a theoretical circular home range of the respective area.

In reality, it is likely that only some males and females respond, with females showing less responsiveness to a playback of a territorial call compared to males (Lin et al., 2007). This leads to an underestimation of the number of pairs and an overestimation of the home range size. Additionally, breeding home ranges used by foraging birds may not always be adjacent to each other, and areas of unoccupied habitat might separate them, further contributing to an overestimation of the home range size. However, if some of the detected pairs have home ranges that extend beyond the transect area (i.e., more than 100 m away from the transect route), or if some pairs did not respond to the playback at all, the values may underestimate the home range size. Unfortunately, no further information is available to determine the extent of these effects. However, we believe that overestimation is likely in these calculations based solely on density, and that the estimate of ~ 20 ha is closer to reality. This value is similar to the estimate based on bird foraging behavior during nestling provisioning (as mentioned in 1 above).

3) Finally, we browsed the literature on the breeding territory/home range of other insectivorous species. Among Amazonian terrestrial insectivorous birds (Stouffer, 2007) of body sizes that are mostly smaller than the Fairy Pitta, the average territory sizes varied widely from 6 to 25.5 ha (e.g., *Sclerurus mexicanus*, 25 g, 25.5 ha; *S. caudacutus*, 38.9 g, 21.3 ha; *Myrmornis torquata*, 44.6 g, 16.0 ha; *Formicarius colma*, 46 g, 6.6 ha; *Formicarius analis*, 62.2 g, 12 ha). For birds of similar body size to the Fairy Pitta (range 70–136 g), the estimates of average breeding territory sizes varied from 6 to over 35 ha in the Amazonian bird community (Terborgh et al., 1990; examples: *Dendrexetastes rufigula*, 70 g, 13 ha; *Turdus hauxwelli*, 72 g, 6 ha; *Dendracolaptes certhia*, 73 g, 15 ha; *Celeus grammicus*, 79 g, 35 ha; *Dendracolaptes picumnus*, 80 g, 21 ha; *Chamaeza nobilis*, 123 g, 30 ha; *Xiphocolaptes promeropirhynchus*, 136 g, ≥ 35 ha). The largest member of the terrestrial insectivores' guild within its Amazonian range, the Variegated Antpitta (*Grallaria varia*) with body length similar to the Fairy Pitta, has been reported to have a home range of 17.6 ha based on radio telemetry (Jirinec et al., 2018). Finally, an African thrush (*Zoothera roehli*) of body size (66.5–75.5 g) slightly smaller than the Fairy Pitta has individual home range sizes measured by radio-telemetry ranging from 3.8 to 27.5 ha in primary and moderately disturbed forests (Newmark, Mkongewa & Sobek, 2010). Among these estimates, the smaller values may result from a smaller number of location points, a shorter monitoring period, or both. It is well known that home range size estimates increase with the number of location points sampled and the duration of the sampling period.
Based on these three approaches, and considering that the generally smaller body sizes of the other species mentioned above might have contributed to smaller territories/home ranges than expected for the Fairy Pitta, we decided to use in our calculations the "observed home range size" range of 10–30 ha. We also checked that our conclusions would be similar if we had used the range 5–20 ha or 10–20 ha, albeit the exact numerical values of the overlap (see Methods part 10) between observed and predicted home range size would be different.

References cited in Supplementary Methods

Jiang A, Yang G, Pagani-Núñez E, Jiang D. 2017. Ecology of two Pittas (Pitta soror and Pitta nympha) in limestone forests of South China. *Journal of Natural History* 51:1929–1941. DOI: 10.1080/00222933.2017.1355490.

Jirinec V, Elizondo EC, Rutt CL, Stouffer PC. 2018. space use, diurnal movement, and roosting of a variegated ant-pitta (Grallaria varia) in central Amazonia. *Ornitologia Neotropical* 29:13–20. DOI: 10.58843/ornneo.v29i1.325.

Kim E-M. 2010. *The Fairy Pitta, comparison of domestic and foreign research status. In Open Seminar Material Collection, Korea Forest Research Institute, Research data No. 386.*

Lin R, Lee P, Ding T, Lin YK. 2007. Effectiveness of Playbacks in Censusing the Fairy Pitta (Pitta nympha) during the Breeding Season in Taiwan. *Zoological Studies* 46:242–248.

Newmark WD, Mkongewa VJ, Sobek AD. 2010. Ranging behavior and habitat selection of terrestrial insectivorous birds in north-east Tanzania: Implications for corridor design in the Eastern Arc Mountains. *Animal Conservation* 13:474–482. DOI: 10.1111/j.1469-1795.2010.00366.x.

Okada M. 1999. Fairy Pitta at the environs of River Shimantogawa. *Birder* 13:54–57.

Stouffer PC. 2007. Density, territory size, and long-term spatial dynamics of a guild of terrestrial insectivorous birds near manaus, Brazil. *The Auk* 124:291–306.

Terborgh J, Robinson SK, Theodore, A. Parker III Charles AM, Pierpont N. 1990. Structure and organization of an amazonian forest bird community. *Ecological Monographs* 60:213–238. DOI: 10.2307/1943045.

Papers cited in BirdLife International 2001; Kim 2010; Kim 2014

Fujida, T., Okeguchi, H. & Sawada, Y. *Ecology and distritbution of the Indian Pitta Pitta brachyura . Wild Bird Society of Japan, Special Bird Survey Report [in Japanese; 藤田剛, 桶口廣芳, 澤田佳長. 1992. 日本にお けるの ヤイロチョウ Pitta brachyura の生態 分布. 日本野鳥の會, 特殊鳥類調査報告書, pp.1-8.]*. (1992).

Okada, M., 1999. Fairy Pitta at the environs of River Shimantogawa. Birder 13, 54–57.

Kim, H. Ecology of the fairy pitta. Korean. Cultural Research Institute 5, 235–240. [in Korean: 김헌규. 팔색조의 생태. 한국문화연구원논총 5, 235–240.] (1964).
